# PSMA PET–guided intensification of postprostatectomy salvage radiotherapy for prostate cancer: a systematic review and meta-analysis

**DOI:** 10.3389/fonc.2026.1779689

**Published:** 2026-03-10

**Authors:** Geng Liu, Yuanyuan Shu, Jiaobao Hong, Zhe Li, Xiaohua Yang

**Affiliations:** Department of Urology Surgery,The First People’s Hospital of Jiande, Jiande, China

**Keywords:** biochemical recurrence, prostate cancer, PSMA PET, salvage radiotherapy, treatment intensification

## Abstract

**Background:**

Prostate-specific membrane antigen (PSMA) PET is used to guide postprostatectomy salvage radiotherapy (SRT) and enable intensification through dose escalation and target modification. The oncologic benefit and safety profile of PSMA PET–guided intensification remain uncertain. We aimed to synthesize comparative and single-arm evidence on oncologic outcomes and toxicity of PSMA PET–guided intensification of postprostatectomy SRT.

**Methods:**

We performed a systematic review and meta-analysis following PRISMA 2020 guidelines. PubMed, Web of Science, Scopus, Embase and Cochrane Library were searched from inception to 26 December 2025. Comparative and single-arm studies evaluating PSMA PET–guided intensification of postprostatectomy SRT were included. We included clinical studies using PSMA PET/CT or PET/MRI to guide radiotherapy intensification and excluded preclinical studies and non-original reports. Primary outcomes were failure-free survival (FFS) and biochemical recurrence–free survival (bRFS). Secondary outcomes included metastasis- and survival-related endpoints, treatment escalation, and toxicity. Meta-analysis was conducted only when sufficient comparative data were available. Hazard ratios were pooled in RevMan 5.4.1 using fixed- or random-effects models according to heterogeneity. Risk of bias was assessed with the Newcastle–Ottawa Scale. Statistical significance was set at two-sided P<0.05.

**Results:**

Fifteen studies met inclusion criteria, including five comparative and ten single-arm studies. Two studies reported FFS-type endpoints; because only two studies were available and endpoint definitions differed substantially, these findings were summarized descriptively. Four comparative studies involving 692 patients contributed to bRFS meta-analysis. PSMA PET–guided SRT showed numerically improved bRFS versus standard SRT, but the difference was not statistically significant (pooled HR 0.61, 95% CI 0.33–1.13; P = 0.12; I²=55%). Other secondary oncologic outcomes were variably reported with limited events and were synthesized descriptively. Severe genitourinary or gastrointestinal toxicity was uncommon, and some studies suggested delayed treatment escalation.

**Conclusions:**

PSMA PET–guided intensification of postprostatectomy SRT may improve biochemical control without an increase in severe toxicity; however, a statistically significant bRFS benefit was not demonstrated and evidence for other oncologic outcomes remains limited.

**Systematic review registration:**

https://www.crd.york.ac.uk/prospero/, identifier CRD420261277044.

## Introduction

Prostate cancer remains a major global health burden; in 2022, an estimated 1.5 million new cases and 397,000 deaths occurred worldwide (1). Biochemical recurrence after radical prostatectomy remains common and clinically consequential, even in the contemporary era. For men with rising prostate-specific antigen (PSA) following prostatectomy, salvage radiotherapy is a potentially curative option and is favored over routine adjuvant radiotherapy in many patients, given randomized evidence showing comparable cancer control with a policy of early salvage while reducing treatment-related morbidity (2–4). Accordingly, contemporary practice emphasizes timely, risk-adapted salvage radiotherapy, guided by clinicopathologic risk features and PSA kinetics (5, 6).

In contemporary practice, postprostatectomy salvage radiotherapy is most often delivered with image-guided external-beam techniques to the prostate bed alone or with elective pelvic nodal irradiation, with short-term androgen deprivation therapy considered in selected higher-risk settings (5). For macroscopic local recurrence, focal dose escalation using a boost or simultaneous integrated boost is commonly applied when feasible, and metastasis-directed radiotherapy may be considered in carefully selected patients with limited metastatic burden (5, 7).

A major limitation of conventional salvage radiotherapy is uncertainty regarding disease location at low PSA levels. Conventional imaging may fail to identify occult local, nodal, or distant lesions, risking geographic miss and undertreatment. Prostate-specific membrane antigen positron emission tomography (PSMA PET) has transformed the diagnostic landscape by improving detection and localization of recurrent prostate cancer, including at low PSA values, with high lesion-level validation in prospective datasets (8, 9). Moreover, PSMA PET has demonstrated superior accuracy compared with conventional imaging, supporting its integration into modern staging and restaging pathways (10).

Beyond detection, PSMA PET can meaningfully alter radiotherapy intent and technique. Compared with alternative PET tracers, PSMA PET has shown improved lesion detection for biochemical recurrence, which directly informs target delineation and may justify treatment intensification through dose escalation to PET-avid lesions or expansion to elective nodal volumes (11). These imaging-driven adaptations are increasingly reflected in guideline recommendations that endorse PSMA PET for evaluation of postprostatectomy recurrence and encourage treatment individualization based on imaging and risk stratification (5, 6).

However, whether PSMA PET–guided intensification translates into improved oncologic outcomes without compromising safety remains uncertain. The available evidence spans heterogeneous designs including comparative cohorts, single-arm series, and emerging randomized data evaluating PSMA PET–guided intensification strategies in the salvage setting (7). Differences in intensification approaches, endpoint definitions, and follow-up duration further complicate inference. Therefore, we performed a systematic review and meta-analysis to synthesize the oncologic effectiveness and toxicity of PSMA PET–guided intensification of postprostatectomy salvage radiotherapy, incorporating both comparative and single-arm evidence and prioritizing time-to-event outcomes when reported.

## Methods

This systematic review and meta-analysis was prospectively registered in the International Prospective Register of Systematic Reviews (PROSPERO) (CRD420261277044) and conducted in accordance with the Preferred Reporting Items for Systematic Reviews and Meta-Analyses (PRISMA) 2020 guidelines (12) and the methodological recommendations of the Cochrane Handbook for Systematic Reviews of Interventions (13). A completed PRISMA 2020 checklist is provided in the **Supplementary Materials**. Key procedures included systematic literature searches, dual independent screening for study inclusion, standardized data extraction, and structured synthesis to ensure methodological consistency and reproducibility.

### Search strategy

A comprehensive literature search was performed by two independent investigators in PubMed, Web of Science, Scopus, Embase and Cochrane Library from database inception to December 26, 2025. Search terms combined concepts related to prostate cancer, PSMA-targeted PET imaging, and salvage radiotherapy, including (“prostate cancer” OR “prostatic carcinoma”) AND (“PSMA PET” OR “PSMA PET/CT” OR “PSMA PET/MRI” OR “^68^Ga-PSMA” OR “^18^F-PSMA” OR “DCFPyL” OR “PSMA-1007”) AND (“salvage radiotherapy” OR “salvage radiation therapy” OR “postprostatectomy radiotherapy”). Reference lists of included studies and relevant reviews were manually screened to identify additional eligible studies. Only English-language articles were considered. The full search strategies for all databases are provided in **Supplementary Appendix S1**.

### Inclusion and exclusion criteria

Randomized controlled trials (RCT), prospective studies, and retrospective studies with either comparative cohorts or single-arm designs were eligible. Studies were included if they met all of the following criteria: (1) patients with histologically confirmed prostate cancer who previously underwent radical prostatectomy; (2) patients receiving salvage radiotherapy for biochemical recurrence and/or PSA persistence; (3) PSMA-targeted PET imaging performed before salvage radiotherapy planning, including PET/computed tomography (PET/CT) or PET/magnetic resonance imaging (PET/MRI) using any PSMA tracer; (4) PSMA PET–guided radiotherapy intensification that was clearly identifiable, defined as at least one of the following strategies: focal dose escalation or boost, simultaneous integrated boost (SIB) to PSMA-avid lesions, expansion of target volumes from prostate bed only to pelvic nodal irradiation or whole-pelvis radiotherapy (WPRT) based on PSMA PET findings, or lesion-directed radiotherapy targeting PSMA PET–positive disease; and (5) reporting at least one outcome of interest, including biochemical recurrence–free survival (bRFS), failure-free survival (FFS), metastasis-free survival (MFS), distant metastasis–free survival (DMFS), disease-free survival (DFS), event-free survival (EFS), overall survival (OS), and prostate cancer–specific survival (PCSS), and/or treatment-related toxicity.

Studies were excluded if they met any of the following criteria: (1) primary definitive treatment was radiotherapy rather than radical prostatectomy; (2) the cohort represented re-irradiation of the prostate bed or pelvic region; (3) radiotherapy was delivered with palliative intent for widespread metastatic disease rather than salvage or curative intent; (4) patients received only systemic therapy without any salvage radiotherapy; (5) publication type was a case report, review, editorial, conference abstract, or other non-original research; (6) full text was unavailable or inaccessible; (7) data were incomplete, unusable, or clearly erroneous for analysis; (8) studies had severe methodological bias that precluded meaningful interpretation; or (9) duplicate or overlapping publications were identified, in which case the most complete dataset was retained. A two-stage screening process was performed independently by two reviewers, including deduplication and title and abstract screening followed by full-text eligibility assessment, with disagreements resolved by consensus or adjudication by a third reviewer.

### Data extraction

Data extraction was conducted independently by two reviewers using a standardized form. Extracted variables included first author, year, country or region, study design, sample size, and follow-up; baseline patient and disease characteristics including PSA values at imaging and or radiotherapy when reported, pathologic stage, nodal status, margin status, Gleason or ISUP grade, and adverse pathologic features; prior post-prostatectomy treatments including androgen deprivation therapy (ADT); imaging modality and tracer; and radiotherapy details including target volumes, dose prescription, and the specific intensification strategy used. Outcomes of interest included failure-free survival and biochemical recurrence–free survival as primary endpoints, and secondary endpoints including MFS or DMFS, OS, prostate cancer–specific survival, treatment escalation–related outcomes such as ADT-free survival or time to subsequent therapy when available, and acute and late genitourinary (GU) and gastrointestinal (GI) toxicities graded using the Common Terminology Criteria for Adverse Events (CTCAE) version 4.0 or 5.0. When available, hazard ratios (HRs), odds ratios, and corresponding 95% confidence intervals (CIs) were extracted directly from the reports; discrepancies were resolved through discussion with a third investigator to ensure accuracy and consistency.

### Assessment of bias

Risk of bias for non-randomised studies was assessed using the Newcastle–Ottawa Scale (NOS), which evaluates study quality across three domains: selection, comparability and outcome/exposure (maximum 9 points). In line with contemporary practice, studies scoring ≥5 points were considered to have at least moderate methodological quality. Two independent appraisers conducted quality assessments using this structured instrument, and inter-rater discrepancies were resolved through consensus discussions moderated by a third reviewer to ensure standardized interpretation of methodological rigor.

### Statistical analysis

Quantitative synthesis was undertaken only when sufficient comparative data were available. Biochemical recurrence–free survival (bRFS) was the sole endpoint eligible for meta-analysis. Meta-analyses were conducted using Review Manager (RevMan) version 5.4.1. HRs and 95% CIs were converted to log(HR) and standard error values prior to pooling. Heterogeneity was assessed using the I² statistic and Cochrane’s Q test (α=0.10). Fixed-effects models were applied when heterogeneity was low (I² ≤50% and P≥0.10), whereas random-effects models were used for substantial heterogeneity (I² >50% and P<0.10). Sensitivity analyses were performed by sequentially omitting each study to evaluate the robustness of pooled estimates. Other secondary oncologic outcomes (MFS/DMFS, OS/PCSS), toxicity endpoints, and treatment escalation–related outcomes were synthesized descriptively due to heterogeneous outcome definitions and limited event numbers. Statistical significance was set at two-sided P<0.05. Publication bias was assessed qualitatively; formal tests and funnel plots were not performed because fewer than 10 studies were available.

## Results

### Systematic review

#### Study selection and overall characteristics

**Figure 1:**
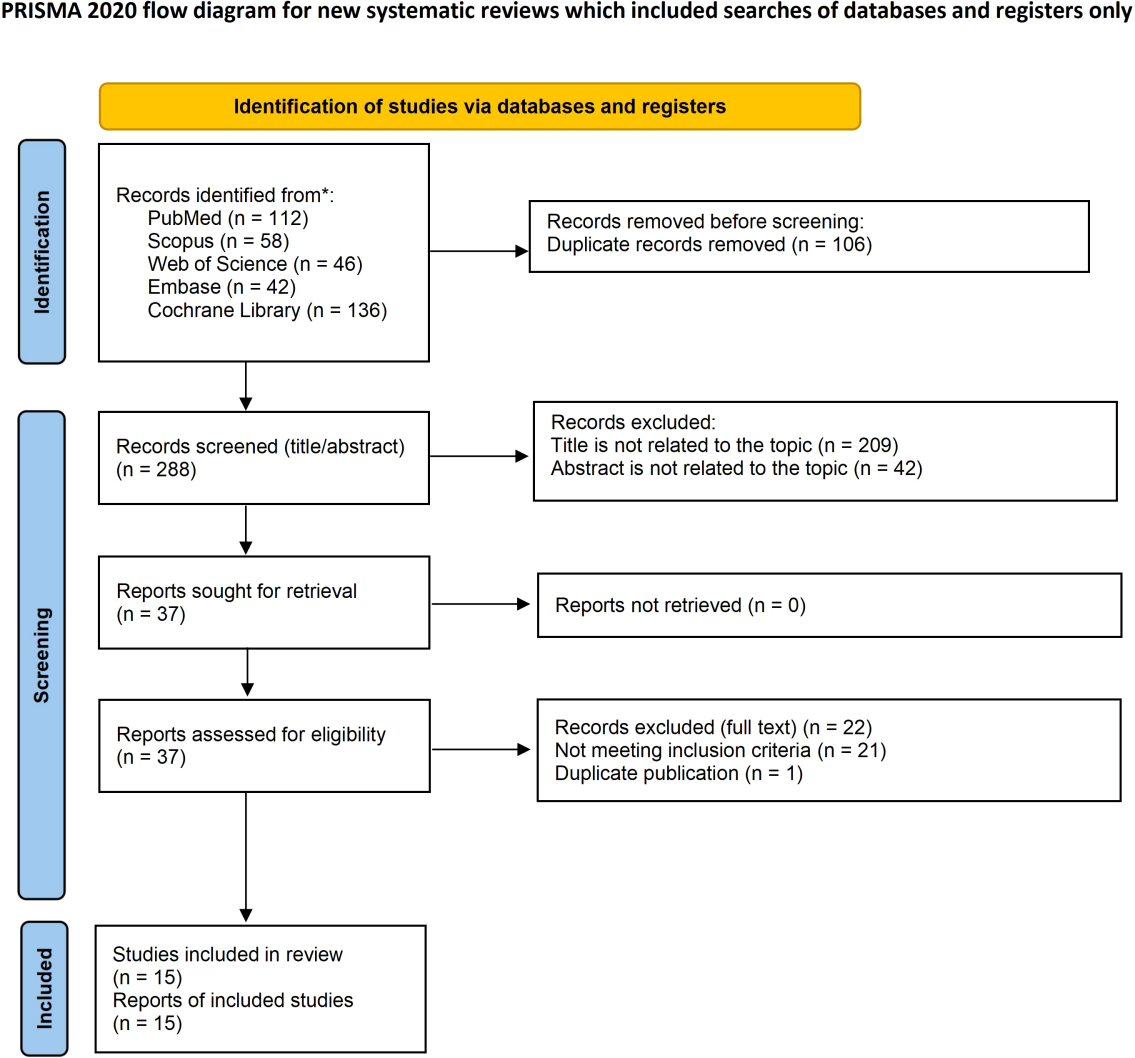
The PRISMA flow diagram displays the details of the selection process. *From: Page, M.J., McKenzie, J.E., Bossuyt, P.M., Boutron, I., Hofmann, T.C., Mulrow, C.D., Shamseer, L., Tetzlaf, J.M., Akl, E.A., Brennan, S.E., et al. (2021). The PRISMA 2020 statement: an updated guideline for reporting systematic reviews. Syst Rev. Mar 29;10(1):89. doi: 10.1186/s13643-021-01626-4. PMID: 33781348; PMCID: PMC8008539.

The electronic search from database inception to 26 December 2025 yielded 394 records; after de-duplication and a two-phase screening process, 15 studies met the predefined eligibility criteria and were included in the qualitative synthesis (**Figure 1**) (7, 14–27). Across the included studies, a total of 1,892 patients received postprostatectomy salvage radiotherapy (one salvage RT course per patient in most studies; fraction number was not consistently reported). These comprised five comparative studies (7, 14, 20, 25, 27) and ten single-arm studies (15–19, 21–24, 26) evaluating PSMA PET–guided intensification of postprostatectomy salvage radiotherapy in patients treated for biochemical recurrence and/or PSA persistence. Study designs included a RCT as well as prospective and retrospective cohort studies, with substantial variability in imaging tracers and radiotherapy intensification strategies, including focal dose escalation or SIB to PSMA-avid lesions, elective nodal irradiation or WPRT, and lesion-directed radiotherapy approaches. Follow-up durations ranged from short-term assessments focused on PSA response to mid-term outcomes reported at 2 to 3 years, and outcome definitions were heterogeneous across studies. Median follow-up ranged from 6.9 to 44.4 months among studies reporting it; three studies did not report median follow-up. Because not all studies reported every outcome of interest, the number of studies and patients contributing to each analysis differed across endpoints. Quantitative meta-analysis was feasible only for biochemical recurrence–free survival, whereas failure-free survival and other secondary oncologic and toxicity outcomes were synthesized descriptively due to sparse reporting and heterogeneity in endpoint definitions. Key study and patient characteristics are summarized in **Tables 1A**, **1B**, with detailed treatment characteristics and outcome data provided in **Supplementary Table S1A–D**. Risk-of-bias assessments for non-randomized studies using the Newcastle–Ottawa Scale indicated overall moderate-to-high methodological quality and are presented in **Table 2**.

**Table 1A:** Study characteristics.

Study (year)	Country/Region	Study design	Comparative type	Clinical setting	Comparator/control arm	Total N	N (PSMA-iSRT)	N (Control)	Baseline PSA at PET	Baseline PSA at SRT	Median follow-up (months)
Arifin et al. (2023) (14)	Canada (London, ON)	Single-center retrospective cohort study	Propensity score matching (1:1) + full cohort analysis	Post-RP biochemical failure; salvage EBRT (early vs late salvage: PSA <0.5 vs 0.5–1.5)	Salvage RT without PSMA-PET prior to RT (33 fx search; no PSMA-PET access)	124 (PSMA 44; control 80); matched 68 (34 vs 34)	44 (matched 34)	80 (matched 34)	Pre-RT PSA median (IQR): 0.48 (0.26–0.73) vs 0.20 (0.14–0.28); matched 0.34 vs 0.21	Same as pre-RT PSA (interval PET→RT NR)	Matched 26 (IQR 18.8–33)
Petit et al. (2025) (7)	Canada	Bi-institutional prospective observational cohort study	Parallel 1:1 randomized	Post-RP BCR; planned salvage RT (eligibility PSA >0.1 ng/mL; delayed SRT: median ~19.5–19.9 mo from surgery)	Standard-of-care SRT (prostate bed ± elective pelvic nodes; no PSMA-PET–guided intensification)	130 randomized; 128 treated	64	64	0.3 ng/mL (0.1–2.4) (experimental arm; enrollment; PET baseline)	NR (SRT PSA not separately reported; control enrollment PSA 0.3 [0.1–3.0])	37 (range 7–60) months
Bluemel et al. (2016) (15)	Germany	Bi-institutional retrospective cohort study	Single-arm (no external control; PET changed vs confirmed plan only)	PSA persistence (16/45) or BCR (29/45); planned post-RP SRT to prostate bed	None (all were intended for prostate-bed SRT before PET)	45	19 (PET-led change/intensification recommendation; not randomized)	26 (plan confirmed; not a true control)	0.67 ng/mL (range 0.10–11.22)	NR (subset treated+FU n=21: 0.60 ng/mL; range 0.10–3.51)	6.92 months (range 1.15–24.36) in subset with follow-up (n=21)
Dhere et al. (2025) (16)	USA	Single-center prospective observational cohort study	Parallel two-arm RCT (18F-fluciclovine PET/CT–guided RT vs 68Ga-PSMA-11 PET/CT–guided RT)	Post-RP detectable PSA (biochemical recurrence/PSA persistence not separated); some treated before PSA 0.2 ng/mL threshold	18F-fluciclovine PET/CT–guided post-prostatectomy RT (not standard SRT without PET)	119 (received RT on study; 140 randomized)	60	59	NR (PET-time PSA not separately reported); pre-radiation PSA median: PSMA 0.35 (0.18–0.74), Fluciclovine 0.27 (0.14–0.74) ng/mL	Pre-radiation PSA: PSMA 0.35 (0.18–0.74), Fluciclovine 0.27 (0.14–0.74) ng/mL	Acute toxicity assessed ≤90 days post-RT (no median follow-up reported)
Gunnlaugsson et al. (2022) (17)	Sweden	Single-center prospective phase II observational cohort study	Single-arm adaptive; internal comparison (responders vs non-responders)	BCR after RP; confirmatory PSA ≥0.15 ng/mL; SRT initiated at low PSA (median 0.25)	No external control. Internal subgroup: responders PB-only SRT vs non-responders intensified SRT (PB+LNI ± PET-directed boost).	97 analyzed (100 enrolled; 2 withdrew; 1 excluded per protocol)	63 (non-responders; intensified SRT)	34 (responders; PB-only SRT)	NR	0.25 (0.19–0.37) ng/mL	38 (IQR 29–48)
Janbain et al. (2024) (18)	Germany; Cyprus; Australia; Italy; Switzerland	Multicenter retrospective cohort study	Single-arm (no comparator); predictive modeling	Post-RP PSA persistence or recurrence; PSA ≥0.1 ng/mL; sRT after PSMA-PET	None	1029	1029	0	NR (only PSA before sRT categories reported)	NR (categories: 0.01–0.2 23.9%; >0.2–0.5 37.4%; >0.5–1 16.7%; >1 22.0%)	NR (model performance evaluated over 12–85 months horizon)
Jani et al. (2025) (19)	USA	Single-center retrospective cohort study	Parallel RCT: [18F]-fluciclovine PET/CT–guided SRT (Arm 1) vs [68Ga]-PSMA-11 PET/CT–guided SRT (Arm 2); primary comparison vs EMPIRE-1 historical fluciclovine arm (no PET-dose escalation)	Post-RP detectable PSA/biochemical progression; conventional imaging negative; salvage setting (BCR vs PSA persistence not separated; early/delayed NR)	[18F]-fluciclovine PET/CT–guided SRT with optional PET-directed SIB (not standard SRT without PET)	119 (received RT; 140 randomized)	60	59	PET-time PSA NR; pre-RT PSA (used as closest): Arm 2 0.4 (0.2–0.8) [range 0.03–75.9]; Arm 1 0.2 (0.2–0.8) [range 0.01–19.4]	Pre-RT PSA: Arm 2 0.4 (0.2–0.8) [0.03–75.9]; Arm 1 0.2 (0.2–0.8) [0.01–19.4]	31.2 (IQR 21.6–48.0)
Kirste et al. (2021) (20)	Germany; Switzerland	Multicenter retrospective cohort study	Non-randomized comparative (PDRT vs PDRT+elective RT)	Post-RP oligorecurrent PC (max 5 visceral/bone mets); 68Ga-PSMA PET/CT-positive; curative-intent salvage RT/MDT	PDRT only (RT to PSMA PET-positive lesions only; no elective prostate bed/pelvic/para-aortic RT)	394	190	204	1.2 ng/mL (0.04–47.5) (PSA at time of RT; PET performed pre-RT)	1.2 ng/mL (0.04–47.5)	28 (1–71)
Rogowski et al. (2022) (21)	Germany	Single-center retrospective cohort study	Single-arm retrospective cohort (no comparator)	bcP 76%; bcR 24%; nodal recurrence; timing early/delayed NR (median RP→bcR 22.5 months)	None (single-arm)	NR	NR	NR	Overall 1.4 (0.1–40.1) ng/mL; bcP 1.7 (0.1–40.1); bcR 0.6 (0.3–5.1)	NR (analyzed as PSA <1 vs ≥1 ng/mL before sRT)	NR
Schmidt-Hegemann et al. (2019) (22)	Germany	Bi-institutional retrospective cohort study	Single-arm; historical comparison (non-concurrent) mentioned	Post-RP biochemical recurrence; PSA persistence excluded; distant metastases excluded	None (no concurrent standard SRT control; compared with historical data only)	90	90	0	0.43 ng/mL (range 0.10–6.24) at PSMA PET/CT	0.44 ng/mL (range 0.11–6.24) before RT	23 (range 1–47)
Spohn et al. (2022) (23)	Germany; Italy (Bologna)	Multicenter retrospective cohort study	Single-arm (no external comparator); prognostic factor analysis (SUVmax)	Post-RP patients with PSA persistence and/or biochemical recurrence; PET-positive local recurrence and/or pelvic nodal recurrence; sRT 2014–2020	None (no standard SRT control arm)	235	235	0	NR (patients underwent 68Ga-PSMA11 PET prior to sRT; baseline PSA at PET not reported as median)	Reported as categories pre-sRT PSA: <0.5 ng/mL 66/235 (28%); ≥0.5 ng/mL 164/235 (70%); NA 5/235 (2%)	24 (IQR 16–41)
Tamihardja et al. (2022) (24)	Germany	Single-center retrospective cohort study	Single-arm (no comparator)	Post-RP biochemical relapse; PSMA PET+ macroscopic prostatic fossa recurrence ± locoregional LN; salvage RT (dose-escalated)	None	59	59	0	NR (PSA at PET not separately reported; PSA at SRT start 0.8 [0.4–1.7])	0.8 ng/mL (IQR 0.4–1.7)	38.2 (IQR 29.0–48.3)
Trapp et al. (2024) (25)	Germany/Switzerland/Australia/Cyprus/Italy	Multicenter retrospective cohort study	Propensity score–matched comparative (WPRT vs HPRT)	Post-RP nodal recurrence on PSMA PET/CT; includes PSA recurrence and PSA persistence	Control: HPRT (unilateral hemi-pelvis elective nodal RT) vs Intervention: WPRT (bilateral whole pelvis elective nodal RT); both PSMA PET-guided	102 (propensity-matched final cohort from 273 total)	51 (WPRT)	51 (HPRT)	Reported as categories (PSA before PET/CT): ≤0.2: 1/51 (2%) vs 1/51 (2%); 0.21–0.5: 11 (22%) vs 12 (24%); 0.51–1.0: 13 (26%) vs 9 (18%); >1.0: 26 (51%) vs 29 (57%)	NR	Median follow-up: 29 overall; 40 (14–84) WPRT vs 28 (12–64) HPRT
Fuertes Vallés et al. (2025) (26)	Spain	Single-center retrospective cohort study	Single-arm (no comparator)	Post-RP isolated prostate bed relapse (biochemical recurrence setting); salvage intent	None	16	16	0	0.41 ng/mL (range 0.20–1.06) [before brachytherapy/imaging]	0.41 ng/mL (range 0.20–1.06) [before HDR; EBRT baseline NR]	44.4 months (range 27.6–62.4) [3.7y, 2.3–5.2]
Vogel et al. (2021) (27)	Germany	Single-center retrospective cohort study	Two-arm retrospective comparative (DE-SRT vs C-SRT; non-randomized)	Post-RP biochemical relapse (BCR); PSA nadir <0.1 ng/mL; early/delayed SRT NR	Conventional salvage RT (C-SRT) to prostate bed ± elective pelvic LNs; no SIB (some patients had PSMA PET but received no boost)	199	101	98	Median PSA at recurrence (proxy PSA at PET): 0.32 (0.02–22.00); C-SRT 0.21 (0.02–5.64); DE-SRT 0.45 (0.02–22.00)	Median PSA before RT: 0.44 (0.02–16.02); C-SRT 0.33 (0.02–16.02); DE-SRT 0.52 (0.02–16.02)	Median follow-up 13.6 months (0.4–70.0)

ENRT, elective nodal radiotherapy; LN, lymph node; LND, lymph node dissection; LVI, lymphovascular invasion; MDT, metastasis-directed therapy; miTNM, molecular imaging TNM; NR, not reported; pN, pathological nodal stage; pT, pathological tumor stage; PRO, patient-reported outcome; QoL, quality of life; ^18^F, fluorine-18; ^68^Ga, gallium-68.

**Table 1B:** Imaging and treatment characteristics.

Study (year)	PSMA PET modality	Tracer	PET positivity & pattern	Intensification type	RT fields	RT dose & fractionation	Technique/planning	ADT use n (%)	ADT duration/regimen
Arifin et al. (2023) (14)	PSMA PET (likely PET/CT; NR)	18F-DCFPyL	Bed 2/44 (4.6%); pelvic LN 16/44 (36.4%); pelvic-only disease required	Field escalation (pelvic nodes) ± boost/SIB to PSMA+ targets	Prostate bed only vs prostate bed + pelvic LNs ± boost	EBRT 33 fx (VMAT): PB 66 Gy; pelvic LNs 50.4 Gy; boost 60–70 Gy	VMAT (photon linac); IGRT/planning guideline NR	ADT: 36.4% vs 22.5% (matched 29.4% vs 29.4%)	Median (IQR) months: 6.0 (4.67–6.05) vs 6.03 (5.95–11.99); range ~4 mo–2 y
Petit et al. (2025) (7)	PSMA-PET/CT	NR	PSMA-PET/CT detected new lesions in 33/64 (52%); distribution/miTNM NR	LN boost (aim EQD2–66 Gy): 19 (30%); prostate bed boost (aim EQD2–77 Gy): 15 (23%); MDT RT (aim EQD2–66 Gy): 2 (3%); possible overlap	Prostate bed for all; elective pelvic nodes 35/64 (55%) vs 31/64 (48%); boosts/MDT to PET-positive sites	Prostate bed: mainly 66 Gy/33 fx (± 70/35); pelvic elective (if used): mainly 44 Gy/22 fx (other 42–48 Gy/20–25 fx); boost/MDT doses reported as EQD2 targets (66 or 77 Gy)	NR (technique/IGRT/guidelines not specified in main text/tables)	54/64 (84%) vs 55/64 (86%)	NR
Bluemel et al. (2016) (15)	PET/CT	68Ga-PSMA I&T	PET+ 24/45 (53.3%): local bed 11/45; LN 8/45 (incl pelvic/retroperitoneal); bone 2/45; rectal/soft tissue 2/45; PET-only lesions 15/24 (62.5%)	Boost/dose escalation (bed up to 76 Gy; nodal boost 60–66 Gy) + ENRT/WPRT (pelvic ± retroperitoneal) ± MDT (bone); ADT for polymetastatic	Prostate bed; pelvic nodes (external/internal iliac/obturator→promontory) ± retroperitoneal (to renal vessels); lesion-directed boosts; bone lesions; rectal wall when indicated	Bed 66–70 Gy (SIB+sequential); morph. local rec up to 76 Gy; elective nodes 50.4 Gy (1.8 Gy/fx); nodal SIB 56 Gy (2 Gy/fx)+boost 60–66 Gy; bone 66 Gy	IG-IMRT (6 MV) with daily CBCT; CTV bed per Poortmans; planning CT 3 mm; margins PTV bed 10 mm (SIB 5 mm)	At imaging: 0%; per recommendation: ADT 2/38 (5.6%) among those following panel	NR
Dhere et al. (2025) (16)	PET/CT	68Ga-PSMA-11 (Arm 2); comparator arm uses 18F-fluciclovine	PET uptake rates not reported as detection endpoints; SIB delivered to PET-uptake in prostate bed: 27/60 (PSMA) vs 47/59 (fluciclovine); pelvic nodal SIB: 7/60 vs 9/59	Boost/SIB to PET-uptake (prostate bed and/or pelvic nodes)	Prostate bed alone if pN0 and no pelvic nodal uptake; prostate bed + pelvic nodes if pN+ and/or pelvic nodal uptake	PTVPB 64.8–70.2 Gy (1.8 Gy/fx); PTVPLV 45–50.4 Gy (1.8 Gy/fx); SIB to PET uptake: prostate bed 70.2–76.0 Gy; pelvis 54.0–56.0 Gy	VMAT; Eclipse planning; target definitions per RTOG consensus/atlas; PET deformable registration; CTV→PTV margin 8 mm (6 mm posterior)	ADT intent (enrolled): PSMA 43/70 (61.4%), Fluciclovine 42/70 (60.0%); analyzed-set ADT NR	NR
Gunnlaugsson et al. (2022) (17)	PET/CT	68Ga-PSMA-11	Detection rate 26% (25/97). By site: prostate bed 9% (9/97); pelvic LN 10% (10/97); bone 5% (5/97); bone+liver 1% (1/97).	Adaptive intensification: elective/adjuvant nodal RT (LNI) for PSA non-responders; SIB/boost to PET+ LN; dose-escalation for local recurrence.	PB for all; +elective/adjuvant LN irradiation for non-responders; PET+ LN boost when present.	PB: 70 Gy/35 fx. Adapt after 50 Gy. Non-responders: +LN 50 Gy/25 fx; PET+ LN SIB 60 Gy/25 fx; local recurrence EQD2(α/β=3) 74–78 Gy.	Biologically adaptive plan-on-plan VMAT; 1 fx/day, 5 fx/week.	0 (prior/ongoing ADT excluded); on-study ADT not reported	NR
Janbain et al. (2024) (18)	PSMA-PET (modality not specified; CT referenced)	PSMA ligand (NR specific tracer)	Local recurrence 42.5%; pelvic LNs positive 30.4% (distant mets excluded)	PSMA-PET–guided sRT; occasional integrated boost to local recurrence; elective pelvic lymphatic radiation and nodal dose-escalation; ADT per risk	Prostatic fossa; elective pelvic lymphatics 38.4%; irradiation to PET-positive pelvic LNs 30.8%	EQD2: prostatic fossa <66 Gy 10.0%, 66–70 53.6%, >70 36.4%; elective pelvic lymphatics <50 Gy 30.3%, >50 4.6% (unknown 3.5%); positive pelvic LNs <50 1.5%, 50–60 13.5%, >60 12.4% (unknown 2.4%)	Intensity-modulated, image-guided sRT	325/1029 (31.6%)	<6 mo 65 (23.1%); 6–12 110 (39.2%); >12–24 57 (20.3%); >24 49 (17.4%); unknown 44 (4.3%) (as reported)
Jani et al. (2025) (19)	PET/CT	[68Ga]-PSMA-11 (Arm 2); comparator tracer: [18F]-fluciclovine	PET patterns (subset with PET data): Arm 2 (n=69)—extrapelvic uptake 8 (12%), pelvic LN ± bed 10 (15%), bed-only 24 (35%), no uptake 27 (39%)	PET-guided target selection + dose escalation (SIB) to PET-uptake sites (prostate bed and/or pelvis); no dose escalation when no uptake; no RT when extrapelvic/bone uptake	Prostate bed alone or prostate bed + whole pelvis (if pelvic LN uptake or pN+); PET-uptake SIB to bed and/or pelvic nodes; extrapelvic uptake → no RT	Prostate bed 64.8–70.2 Gy (36–39 fx); pelvic elective 45.0–50.4 Gy (25–28 fx); SIB: prostate bed up to 76.0 Gy; pelvis up to 56.0 Gy; delivered boost median 74.0 Gy (bed) and 55.0 Gy (pelvic nodes)	Photon IMRT; daily IGRT (kV/kV or CBCT); PET-to-planning CT registration; typical CTV-to-PTV expansion ~0.8 cm	Any ADT: Arm 2 44/70 (63%); Arm 1 42/70 (60%); long-term ADT: 11/70 (16%) vs 14/70 (20%)	Typically 6 months (concurrent with RT); long-term ADT per protocol in subset; regimen NR
Kirste et al. (2021) (20)	PET/CT	68Ga-PSMA-11	Local bed 29.4% (116/394); nodal N1 53.6% (211/394); distant mets 34.5% (136/394): M1a 14.3%, M1b 18.1%, M1c 1.8%	PDRT + elective RT (ePBRT/pelvic/para-aortic ENRT) in addition to lesion-directed RT; ± SIB/boost; SBRT possible	Lesion-directed RT to all PSMA+ lesions; elective RT areas could include prostate bed, pelvic lymphatics, paraaortic nodes	Elective PBRT median 66 Gy (47.5–70; 1.8–2 Gy/fx); elective pelvic lymphatics median EQD2 47.5 Gy (36–56); PSMA+ local bed EQD2 71.2 Gy; pelvic LN EQD2 59.4 Gy; paraaortic LN EQD2–55 Gy; SBRT used 9.6%	Conventional fractionated RT (52.0%) ± SIB (33.0%); SBRT (9.6%); EQD2 (α/β=1.5); planning per institution	130/394 (33.0%)	NR
Rogowski et al. (2022) (21)	PET/CT	68Ga-PSMA-11 75%; 18F-PSMA-1007 25%	NR	NR	NR	NR	NR	NR	NR
Schmidt-Hegemann et al. (2019) (22)	PET/CT	68Ga-PSMA (PSMA-HBED-CC)	PET-positive 47% (42/90): fossa only 27% (24/90), pelvic LN only 13% (12/90), both 7% (6/90); PET-negative 53% (48/90)	PSMA PET/CT–guided individualized sRT: boost/dose escalation to PET+ macroscopic fossa recurrence and nodes; pelvic lymphatic pathway irradiation for PET+ nodes; ADT initiation in PET+ cases	Prostatic fossa in 89/90; pelvic lymphatic pathways treated when PET+ pelvic nodes; boosts to PET+ lesions	Local macroscopic tumor 70.0 Gy (67.2–72); prostatic fossa 66 Gy (59.4–70.2); PET+ lymph node 60.76 Gy (54–66); lymphatic pathways 50.4 Gy (45–50.4); boosts SIB or sequential	IMRT/VMAT with IGRT (2–5×/week); target delineation per RTOG post-op prostate and pelvic LN atlases; 5–7 mm PTV margin	26/90 (28.9%)	ADT/antiandrogen recommended for PET+ lesions (intended 2y); most discontinued after median 5 mo (range 2–23); 4 ongoing at last follow-up
Spohn et al. (2022) (23)	PET/CT	68Ga-PSMA-11	By inclusion: PET-positive pelvic disease only. LR only 97/235 (41%); NR only 95/235 (40%); both 43/235 (18%). LR present 140/235 (60%); NR present 138/235 (59%).	Dose escalation to PET-positive LR/NR + elective lymphatics when nodal disease (PET-guided field/dose intensification)	Prostatic fossa always treated; elective pelvic lymphatics in case of nodal disease (and fossa not omitted even if NR only)	Prostatic fossa/local recurrence dose categorized: <70 Gy 162/235 (69%); ≥70 Gy 33/235 (14%); ≥72 Gy 38/235 (16%); RT to elective pelvics 154/235 (60%). Fractionation NR.	Intensity-modulated sRT (IMRT) (center-specific planning; details NR)	ADT 120/235 (51%)	Among ADT: ≤12 months 71/120 (59%); >12 months 49/120 (41%); specific regimen NR
Tamihardja et al. (2022) (24)	PSMA PET/CT	68Ga-PSMA I&T (74.6%); 18F-PSMA-1007 (25.4%)	Selected PSMA PET+ fossa recurrence (100%); 18.6% with locoregional LN+ (0–3 nodes in 96.6%)	Dose escalation/SIB boost to PET+ local recurrence; SIB boost to PET+ nodes (if present)	Prostate bed + boost to recurrence; ± boost to pelvic LN metastases; elective pelvic nodal irradiation NR	PTV 56.1 Gy/33x1.7; Boost1 69.3 Gy/33x2.1; Boost2 72.6 Gy (IQR 72.6–75.5)/33x2.2 (47.5%); LN boost 66.9 Gy (IQR 61.4–69.3)	IMRT or VMAT; CBCT-guided; RTOG salvage atlas; PET/MRI co-registration (Pinnacle3)	19/59 (32.2%)	Duration 24.2 mo (IQR 15.4–31.0); regimen NR
Trapp et al. (2024) (25)	PSMA PET/CT	68Ga-PSMA or 18F-PSMA	All had PSMA-positive nodal recurrence; local recurrence on PET/CT: 33% vs 6% (WPRT vs HPRT); other patterns NR	ENRT volume comparison (WPRT vs HPRT) with frequent LNM boost (90% vs 86%)	Pelvic lymphatic pathways (whole vs unilateral hemi-pelvis) ± prostate bed RT (78% vs 85%)	Pelvic elective dose (EQD2α/β=1.5): ≤50 Gy 82% vs 86%; >50 Gy 14% vs 14%. LNM dose (EQD2α/β=1.5): ≤50 Gy 2% vs 0%; 50.1–60 Gy 24% vs 86%; >60 Gy 59% vs 0% (WPRT vs HPRT). Fractionation details NR	Technique/IGRT/guideline NR (multi-institutional; no standardized contouring/dose prescriptions stated)	ADT during RT: 35/51 (69%) vs 36/51 (71%)	ADT duration: ≤6 mo 8% vs 4%; >6–≤12 mo 10% vs 16%; >12–≤24 mo 10% vs 4%; >24 mo 2% vs 6%; missing 71% both arms
Fuertes Vallés et al. (2025) (26)	PSMA PET/CT (plus mpMRI; lesion visible on TRUS)	NR	Detection/eligibility: 100% local IPBR; PSMA+/MRI+ 75%, PSMA+/MRI− 25%; no nodal/distant disease by exclusion; miTNM NR	Dose escalation via HDR brachytherapy boost + EBRT to prostate bed + elective pelvic nodes (combo intensification)	GTV/CTV HDR boost (IPBR) + prostate/seminal vesicle bed (PTV2) + elective pelvic nodes (PTV3: external/internal iliac, obturator)	HDR 19 Gy in 2 fractions to CTV (two consecutive days) + EBRT: PTV2–42 Gy/15 fx; PTV3–40 Gy/15 fx	HDR under TRUS with MRI/TRUS fusion + intraoperative MRI; EBRT with VMAT/IMRT; daily CBCT; fiducials	16/16 (100%)	6 months LHRH agonist; antiandrogen 10 days prior to LHRH initiation
Vogel et al. (2021) (27)	PSMA PET/CT or PET/MRI (EANM/SNMMI guidelines)	[68Ga]PSMA-11; [18F]rhPSMA-7; [18F]rhPSMA-7.3; [18F]PSMA-1007	DE-SRT PSMA PET results: local recurrence 58/101; LN mets 18/101; both 25/101	Boost/SIB to PSMA PET–positive local recurrence and/or lymph nodes (dose-escalation)	C-SRT: PB 68 Gy ± ePLNs; DE-SRT: PB 68 Gy with SIB to local recurrence and/or nodal targets; optional ePLNs with/without SIB	PB 68 Gy/2.0 Gy fx; ePLNs 50.4 Gy/1.8 Gy fx; SIB local recurrence 76.5 Gy/2.25 Gy fx; SIB PET+ LNs 58.8–61.6 Gy/2.1–2.25 Gy fx	IMRT (VMAT or helical IMRT); daily online imaging IGRT; target delineation per RTOG/EORTC; PTV margin 5–10 mm for SIB	Additive ADT: 40/199 (20.1%); C-SRT 12/98 (12.2%); DE-SRT 28/101 (27.7%)	Disease-free survival (DFS) + PSA response

ENRT, elective nodal radiotherapy; LN, lymph node; LND, lymph node dissection; LVI, lymphovascular invasion; MDT, metastasis-directed therapy; miTNM, molecular imaging TNM; NR, not reported; pN, pathological nodal stage; pT, pathological tumor stage; PRO, patient-reported outcome; QoL, quality of life; ^18^F, fluorine-18; ^68^Ga, gallium-68.

**Table 2:** The Newcastle–Ottawa Scale (NOS) for assessing the quality of non-randomised studies included in this review.

Study	Year	Country	Type of Article	The Newcastle-Ottawa Scale (NOS)		
				Selection	Comparability	Exposure
Arifin et al. (14),	2023	Canada	Single-center retrospective cohort study	***	**	***
Petit et al. (7),	2025	Canada	Bi-institutional prospective observational cohort study	***	**	***
Bluemel et al. (15),	2016	Germany	Bi-institutional retrospective cohort study	***	–	**
Dhere et al. (16),	2025	USA	Single-center prospective observational cohort study	***	**	***
Gunnlaugsson et al. (17),	2022	Sweden	Single-center prospective phase II observational cohort study	***	–	***
Janbain et al. (18),	2024	Germany; Cyprus; Australia; Italy; Switzerland	Multicenter retrospective cohort study	***	–	**
Jani et al. (19),	2025	USA	Single-center retrospective cohort study	***	**	***
Kirste et al. (20),	2021	Germany; Switzerland	Multicenter retrospective cohort study	***	*	***
Rogowski et al. (21),	2022	Germany	Single-center retrospective cohort study	***	–	**
Schmidt-Hegemann et al. (22),	2019	Germany	Bi-institutional retrospective cohort study	***	–	***
Spohn et al. (23),	2022	Germany; Italy (Bologna)	Multicenter retrospective cohort study	***	–	***
Tamihardja et al. (24),	2022	Germany	Single-center retrospective cohort study	***	–	**
Trapp et al. (25),	2024	Germany/Switzerland/Australia/Cyprus/Italy	Multicenter retrospective cohort study	***	**	***
Fuertes Vallés et al. (26),	2025	Spain	Single-center retrospective cohort study	***	–	**
Vogel et al. (27),	2021	Germany	Single-center retrospective cohort study	***	**	***

*:1 point.

“– “ indicates that the comparability domain was not applicable because the study was single-arm or lacked an external comparison cohort, and therefore could not be assessed according to the cohort Newcastle–Ottawa Scale.

### Primary outcomes

#### Failure-free survival

Two studies (14, 27) reported FFS-type outcomes, including one randomized trial reporting FFS and one retrospective study reporting disease-free survival (DFS). Because only two studies were available and the endpoint definitions and clinical contexts were not sufficiently comparable, quantitative pooling was not performed and results were summarized descriptively.

In the randomized trial by Arifin et al. (14), PSMA PET–guided intensification of salvage radiotherapy significantly improved FFS compared with standard salvage radiotherapy (HR 0.46, 95% confidence interval [CI] 0.22–0.99).

In contrast, the retrospective study by Vogel et al. (27) reported inferior DFS among patients receiving PSMA PET–based dose-escalated salvage radiotherapy compared with conventional salvage radiotherapy (HR 2.22, 95% CI 1.08–4.58).Notably, these two studies differed substantially in study design, patient selection, and treatment strategies, which likely contributed to the observed heterogeneity.

### Secondary oncologic outcomes

#### Metastasis-free survival/distant metastasis–free survival

Across the included studies, metastasis-related outcomes were variably reported, and quantitative pooling was not feasible because of heterogeneous endpoint definitions and limited event numbers. In a matched cohort comparing PSMA PET–guided salvage radiotherapy with a non–PSMA PET comparator, metastasis-related outcomes were sparsely reported and events were infrequent; therefore, MFS could not be robustly compared between groups (14).

In patients with PSMA PET–positive nodal recurrence treated with salvage elective nodal radiotherapy, the reported 2- and 3-year DMFS rates were 79% and 66%, respectively, whereas longer-term survival outcomes were not assessable given the follow-up duration (22).

Favorable metastatic control was also reported in selected cohorts treated with PSMA PET–guided dose-escalated salvage radiotherapy for macroscopic local recurrence. In one multicenter retrospective analysis, the 3-year MFS rate reached 96.2% (95% CI, 91.2–100.0), suggesting excellent distant disease control in carefully selected patients (24).

#### Overall survival/prostate cancer–specific survival

OS and PCSS were inconsistently reported across studies and were generally immature because of short follow-up and a low number of death events. In the matched cohort study, neither OS nor cancer-specific survival could be evaluated, as no deaths occurred during follow-up (14).

Similarly, studies focusing on PSMA PET–guided salvage radiotherapy for nodal recurrence or macroscopic local relapse noted that available follow-up was insufficient to allow meaningful assessment of OS or PCSS (22, 24).

Taken together, current evidence does not permit reliable estimation of long-term survival benefits associated with PSMA PET–guided intensification strategies, and extended follow-up from prospective and comparative cohorts is warranted.

#### Toxicity and ADT escalation–related outcomes

Treatment-related toxicity and escalation to systemic therapy were key secondary outcomes of interest. Treatment-related toxicity outcomes were reported in 11 of the 15 included studies (acute and/or late GU/GI). Across studies, PSMA PET–guided intensification strategies were generally associated with acceptable toxicity profiles. In cohorts treated with PSMA PET–guided dose-escalated salvage radiotherapy for macroscopic recurrence, the cumulative 3-year incidence of late grade ≥3 GU toxicity was low at approximately 3–4%, and no grade ≥3 GI toxicity was observed (24).

In randomized and prospective imaging-guided studies, acute toxicity was predominantly mild, with no grade ≥3 acute GU or GI events reported in PSMA PET–guided treatment arms (7).

Several studies also reported treatment escalation–related endpoints, such as time to subsequent systemic therapy or event-free survival. Although definitions varied, these outcomes suggested that PSMA PET–guided approaches may postpone subsequent systemic treatment or androgen deprivation therapy initiation in selected patients (7, 14).

### Meta-analysis

#### Biochemical recurrence–free survival

**Figure 2:**
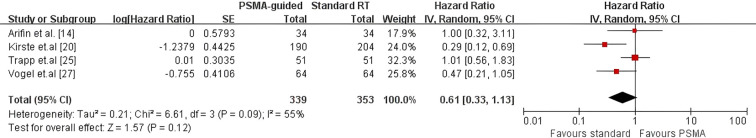
Random-effects meta-analysis of biochemical recurrence–free survival (bRFS) comparing PSMA PET–guided salvage radiotherapy with standard salvage radiotherapy after radical prostatectomy, presented as a forest plot. Hazard ratios (HRs) and 95% confidence intervals (CIs) were pooled using an inverse-variance random-effects model. Squares indicate study-specific effect estimates with sizes proportional to study weight, and the diamond represents the pooled effect estimate. bRFS, biochemical recurrence–free survival; CI, confidence interval; HR, hazard ratio; IV, inverse variance; PSMA, prostate-specific membrane antigen; RT, radiotherapy; SE, standard error.

Four studies encompassing 692 patients were included in the quantitative synthesis of bRFS (14, 20, 25, 27). Compared with standard salvage radiotherapy, PSMA PET–guided radiotherapy was associated with a numerically improved bRFS, although the difference did not reach statistical significance (pooled HR = 0.61, 95% CI 0.33–1.13; *P* = 0.12; random-effects model). Moderate heterogeneity was observed across studies (I² = 55%) (**Figure 2**). Across the four studies included in the bRFS meta-analysis, point estimates generally favored PSMA PET–guided approaches despite variability in study design, patient populations, and biochemical failure definitions.

## Discussion

In this systematic review of 15 studies evaluating PSMA PET–guided intensification of postprostatectomy salvage radiotherapy, imaging-guided escalation was generally feasible and associated with acceptable toxicity. The only outcome suitable for quantitative synthesis showed a numerically improved biochemical recurrence–free survival with PSMA PET–guided strategies, but the pooled comparative difference was not statistically significant. Evidence for other oncologic endpoints remains limited. Failure-free survival–type outcomes were reported in only two studies and were defined differently, while metastasis-free or distant metastasis–free survival and overall or prostate cancer–specific survival were reported inconsistently, with few events and short follow-up. Overall, the current literature supports the clinical rationale for PSMA PET–informed tailoring of salvage radiotherapy, but more mature prospective comparative data are still needed to clarify benefits beyond biochemical control.

A trend toward improved biochemical control with PSMA PET–guided intensification is biologically plausible. Across the included cohorts, PSMA PET findings frequently prompted concrete modifications in target definition and treatment delivery, including focal boosts and/or field expansion (7, 14, 15, 17, 22, 27, 28). This experience also aligns with the broader imaging literature showing that PSMA PET can localize recurrence and meaningfully alter management even at low PSA levels (8, 9, 11, 29, 30). Reflecting this shift, major contemporary pathways now incorporate PSMA PET in the evaluation of postprostatectomy recurrence to support risk-adapted target modification (5, 31, 32). Even so, our pooled comparative estimate for biochemical recurrence–free survival did not reach statistical significance despite a point estimate favoring PSMA PET–guided approaches. This pattern is more consistent with limitations of the current evidence base than with a clear lack of benefit. Only a minority of studies were designed as head-to-head comparisons with harmonized time-to-event reporting, and event numbers were low even within those datasets (7, 14, 27, 28). Many of the remaining studies were single-arm or disease-state–selected cohorts in which PSMA PET primarily served to define targets for escalation rather than enabling a clean comparator contrast (15, 17, 20–25).

Standard salvage radiotherapy is also not a uniform comparator across eras and institutions, which further reduces the contrast a comparative analysis can detect. Even within prospective designs, PSMA PET can shift management in both directions. It can escalate dose and/or volumes when lesions are detected, but it can also redirect intent when unexpected disease distribution is uncovered. As a result, binary comparisons of PSMA PET–guided versus conventional treatment can mix intensification effects with selection effects (7, 28). In the PSMA-SRT randomized phase 3 trial, PSMA PET/CT prompted management changes in roughly one-third of men planned for salvage radiotherapy after radical prostatectomy, underscoring that PSMA PET functions as a decision pathway that reshapes selection and target design rather than a simple add-on test (28). Similarly, in the phase 2 randomized trial of PSMA PET–guided intensification, the PSMA-guided arm used multiple escalation modes, including boosts to bed or nodal targets and, in selected cases, metastasis-directed radiotherapy (11). Together, these features help explain why a directionally favorable bRFS estimate can coexist with a non-significant pooled result in the current literature (7, 14, 27, 28).

The contemporary postprostatectomy radiotherapy paradigm has been shaped by randomized evidence indicating that early salvage radiotherapy can achieve cancer control comparable to routine adjuvant radiotherapy while avoiding overtreatment in many men (2, 3, 33). However, these strategy-defining datasets largely evolved in an era of conventional imaging. In daily salvage decision-making, a practical gap remains: when PSA rises at low levels, clinicians often do not know where the disease resides, and therefore lack a principled basis for adapting volumes and dose when occult nodal or early metastatic disease is present but invisible on standard workup. Randomized data in the salvage setting also show that intensification can matter when appropriately selected. SPPORT (NRG/RTOG 0534) trial demonstrated improved freedom from progression with the addition of pelvic nodal radiotherapy plus short-term ADT to prostate-bed salvage radiotherapy (34). GETUG-AFU 16 trial likewise supported adding short-term androgen suppression to salvage radiotherapy for improved progression-related outcomes (35). These trials establish that what to treat and how much to intensify can change outcomes, but they do not specify how target definition should be tailored once sensitive molecular imaging is integrated into routine care.

PSMA PET has shifted salvage restaging from anatomy-based inference toward biology-informed localization, and this change is apparent across the 15 included studies. PET findings were repeatedly used to redefine targets for lesion-directed boosts, to justify elective nodal coverage in PSMA-positive nodal relapse, or to combine PET-directed and elective fields in oligorecurrent presentations (7, 15, 17, 20–25, 27). In that context, our meta-analysis sits at the junction between the early-salvage strategy era and the molecular-imaging planning era. Current guidelines increasingly endorse PSMA PET for evaluation of postprostatectomy recurrence, while also acknowledging uncertainty about how best to translate PET findings into standardized intensification schemas. The 2024 American Urological Association/American Society for Radiation Oncology/Society of Urologic Oncology (AUA/ASTRO/SUO) salvage therapy guideline provides structured recommendations for treatment delivery in non-metastatic biochemical recurrence and notes that prospective comparative outcome data for imaging-guided radiotherapy adaptation remain limited (31). The 2024 European Association of Urology (EAU) prostate cancer guideline update similarly integrates PSMA PET into pathways for relapsing disease while recognizing that patient selection and standardized intensification approaches are still evolving once PSMA PET reveals nodal or oligometastatic targets (32). Against this backdrop, our synthesis helps frame what the current literature suggests about biochemical outcomes and toxicity, and why longer follow-up and adequately powered comparative studies are needed before specific intensification strategies can be recommended with high confidence (7, 14–17, 20–25, 27).

Beyond treatment planning, PSMA PET may also contribute to response assessment after systemic therapy and/or local radiotherapy by enabling whole-body evaluation of residual PSMA-avid disease and early progression, thereby supporting risk-adapted decisions on consolidation, escalation, or surveillance (36). However, post-therapy interpretation requires caution because androgen-axis therapies can modulate PSMA expression and induce transient uptake changes or flare, while post-radiotherapy inflammatory or reactive uptake may confound lesion-level assessment (37, 38). Moreover, response timing and criteria remain heterogeneous and are not yet uniformly standardized, which limits cross-study comparability and linkage to durable clinical endpoints (39). Future prospective cohorts should predefine modality- and therapy-specific response assessment schedules and criteria and correlate post-treatment PSMA PET response with long-term outcomes such as MFS and treatment-escalation endpoints.

Although biochemical control appears directionally favorable in several PSMA PET–guided intensification cohorts, the available studies are not yet able to support firm conclusions for metastasis-related or survival endpoints. In the matched comparative cohort, metastatic and death events were sparse and time-to-event modeling for OS or PCSS was not feasible; notably, no deaths occurred during follow-up, so survival estimation was not informative (14). Similar constraints apply to prospective and randomized imaging-guided studies, where follow-up remains relatively short and late events are uncommon, leaving metastasis and survival endpoints immature beyond biochemical failure (7, 16, 17). This is also seen in disease-state–selected cohorts. Studies focusing on nodal recurrence or macroscopic local relapse can report early metastasis-related outcomes, but event counts remain limited and observation windows often do not extend long enough for robust survival inference (21, 22, 24, 25). These limitations reflect the natural history of postprostatectomy recurrence. Biochemical relapse typically precedes radiographic metastasis and cancer-specific death by years, and salvage radiotherapy can further delay downstream clinical events. In addition, guideline discussions emphasize substantial variability in endpoint definitions, time origins, and follow-up horizons across relapse settings, which complicates cross-study comparisons (40).

Limited follow-up is only part of the problem. Across the 15 studies, metastasis- and progression-related endpoints are also reported using non-aligned labels and compositions, which limits cross-study comparability and makes pooling unreliable even when estimates trend in the same direction. Metastasis-related outcomes are variably presented as MFS or DMFS and are sometimes reported only as actuarial rates at different time points rather than as harmonized effect estimates (22, 24). Several cohorts report composite outcomes such as FFS or EFS that mix biochemical failure with clinical progression and/or treatment escalation. Others emphasize escalation-sensitive endpoints including time to subsequent systemic therapy, initiation of ADT, ADT-free survival, or castration-resistant prostate cancer–related outcomes, each triggered by distinct events and influenced by practice patterns (7, 14, 18, 20, 21, 25–27). In practical terms, one study may summarize control using 2–3-year MFS or DMFS, another may foreground composite FFS or EFS, and a third may anchor outcomes to ADT initiation or ADT-free survival. These measures address related but distinct questions and should not be treated as interchangeable, particularly given evidence that MFS is a stronger surrogate for OS than PSA-based biochemical endpoints in recurrence settings (41, 42). The current limitation therefore reflects follow-up maturity and inconsistent endpoint reporting within the available PSMA PET–guided salvage radiotherapy literature. It also points to what future cohorts and trials need to standardize: endpoint definitions, time origin, and the parallel reporting of MFS or DMFS alongside treatment-escalation outcomes (7, 14, 16, 17, 25).

Patient selection is likely to be the key determinant of benefit from PSMA PET–guided intensification, and the current literature is best viewed as hypothesis-generating rather than definitive. Across published cohorts, the clearest signal appears in men with PSMA PET–detected nodal recurrence. In this setting, PSMA guidance often leads to elective nodal irradiation with boosts to PET-avid nodes (21, 22, 25, 28). This aligns conceptually with randomized salvage evidence. In SPPORT, adding pelvic nodal radiotherapy plus short-term androgen deprivation therapy improved progression-related outcomes compared with prostate-bed radiotherapy alone (34). GETUG-AFU 16 similarly supports a benefit from adding short-term androgen suppression to salvage radiotherapy (35). Nevertheless, nodal relapse may also reflect a higher likelihood of microscopic spread beyond visible targets. Any apparent advantage of PSMA-guided nodal intensification should therefore be interpreted cautiously and may apply primarily to carefully selected patients treated with comprehensive nodal coverage rather than lesion-only approaches (20–23, 25). A second potentially enriched subgroup is men with macroscopic local recurrence in the prostate bed. In the PSMA era, dose-escalated cohorts focusing on imaging-visible local relapse have reported encouraging metastasis-related outcomes with acceptable toxicity, although prospective comparative confirmation is still needed (24, 26, 27). This approach is also supported by consensus guidance on target volume definition for macroscopic postprostatectomy recurrences, which provides a practical framework for focal boosting in this setting (43). Finally, PSMA PET may have its greatest decision impact in low-volume disease, where identifying a limited number of lesions can redirect management toward lesion-directed boosts or metastasis-directed radiotherapy. This is consistent with phase II evidence that metastasis-directed strategies can delay progression in oligometastatic recurrence, but extrapolation to postprostatectomy salvage intensification should remain cautious and limited to selected patients with limited burden and a realistic chance of durable local-regional control (7, 44–46).

Our study has several strengths. We focused specifically on PSMA PET–guided intensification of postprostatectomy salvage radiotherapy and integrated both comparative and single-arm evidence to reflect contemporary practice. We used a prespecified, clinically grounded definition of intensification that captures dose escalation, target-volume expansion, and lesion-directed strategies. Quantitative synthesis was performed only when comparative data were sufficiently aligned, avoiding inappropriate pooling. Toxicity and treatment-escalation outcomes were summarized alongside oncologic endpoints to support a balanced interpretation of benefits and trade-offs.

The evidence base remains small, with few comparative studies and limited event counts, constraining statistical power and precluding meta-analysis for most endpoints beyond biochemical recurrence–free survival. Substantial clinical and methodological heterogeneity persists across studies, including differences in patient selection, PSMA tracers, radiotherapy techniques, endpoint definitions, and follow-up duration. In addition, the included evidence had limited geographic diversity, with many studies originating from a small number of regions, which may limit generalizability to other practice settings and populations. Imaging modality (PSMA PET/CT vs PET/MRI) was inconsistently reported, limiting our ability to assess whether potentially improved local-recurrence detection translates into better post-SRT outcomes. Most included studies were observational, introducing risks of selection bias and residual confounding despite generally moderate-to-high methodological quality by NOS. Long-term outcomes such as MFS, cancer-specific survival, and overall survival were immature or infrequently reported. Finally, the small number of comparative studies limited any meaningful assessment of small-study effects or publication bias.

In clinical practice, PSMA PET–guided intensification is a reasonable planning strategy for selected postprostatectomy patients undergoing salvage radiotherapy, given its acceptable toxicity profile and a potential improvement in biochemical control, although a statistically significant comparative bRFS benefit has not been demonstrated. However, the current evidence remains largely observational and heterogeneous. Future studies should prioritize adequately powered prospective or randomized cohorts with standardized endpoint definitions and longer follow-up, to clarify effects on metastasis-related outcomes and survival and to identify the subgroups most likely to benefit from imaging-guided dose escalation and target modification.

## Data Availability

The data analyzed in this study is subject to the following licenses/restrictions: Any researchers interested in this study could contact Geng Liu to request the data. Requests to access these datasets should be directed to Geng Liu, 1191662410@qq.com.
