# Supplementary material for: PSMA PET–guided intensification of postprostatectomy salvage radiotherapy for prostate cancer: a systematic review and meta-analysis
Source: Front Oncol. 2026 Mar 10;16:1779689. doi: 10.3389/fonc.2026.1779689 (PMC13008707; doi:10.3389/fonc.2026.1779689)
Supplement: Supplementary file 2 [file DataSheet2.docx]

### Supplementary Methods: Literature search strategy

We systematically searched PubMed, Web of Science Core Collection, Scopus, Embase, and the Cochrane Library for studies published from database inception to 26 December 2025. Only English-language publications were considered. The detailed search strategies for each database are listed below.

#### 1. PubMed

**Database:** PubMed (MEDLINE)
**Date range:** Database inception to 26 December 2025
**Search fields:** MeSH terms and Title/Abstract

#### ****Step 1: #1****

(prostate cancer* OR prostatic cancer* OR prostate carcinoma* OR prostatic neoplasm*) AND (prostatectom* OR postprostatectom* OR "post-prostatectomy" OR "radical prostatectomy") AND (salvage OR "salvage therapy" OR "salvage radiotherap*" OR "salvage radiation*" OR "postprostatectomy radiotherap*" OR "prostate bed radiotherap*" OR SRT)

#### ****Step 2: #2****

(PSMA OR "prostate-specific membrane antigen") AND (PET OR "PET/CT" OR PET-CT OR "PET/MRI" OR PET-MRI OR "positron emission" OR "positron emission tomography")

#### ****Step 3: #3****

boost* OR "dose escalation" OR escalat* OR intensif* OR "simultaneous integrated boost" OR SIB OR "elective nodal" OR ENRT OR "whole pelvis" OR WPRT OR "pelvic nodal" OR "PET-directed" OR "lesion-directed"

**Final combination:**

**#1** AND **#2** AND **#3** NOT ("case report" OR "case reports" OR review OR "systematic review" OR "meta analysis" OR "meta-analysis" OR metaanalysis)

#### 2. Web of Science Core Collection

**Database:** Web of Science Core Collection
**Search field:** Topic (TS)
**Search date:** up to 26 December 2025

#### ****Step 1:**** S1 = ALL = ( "prostate cancer" OR "prostatic cancer" OR "prostate carcinoma" OR "prostatic neoplasm") AND (prostatectom OR postprostatectom OR "post-prostatectomy" OR "radical prostatectomy") AND ( salvage OR "salvage radiotherap" OR "salvage radiation" OR "postprostatectomy radiotherap" OR "prostate bed radiotherap" OR SRT)

#### ****Step 2:**** S2 = ALL = ( PSMA OR "prostate-specific membrane antigen" ) AND (PET OR "PET/CT" OR PET-CT OR "PET/MRI" OR PET-MRI OR "positron emission" OR "positron emission tomography")

#### ****Step 3:**** S3 = ALL = (boost OR "dose escalation" OR escalat OR intensif OR "simultaneous integrated boost" OR SIB OR "elective nodal" OR ENRT OR "whole pelvis" OR WPRT OR "pelvic nodal" OR "PET-directed" OR "lesion-directed")

**Final combination:**
S4 = S1 AND S2 AND S3 NOT ALL = ("case report" OR "case reports" OR review OR "systematic review" OR "meta-analysis" OR "meta analysis" OR metaanalysis)

#### 3. Scopus

**Database:** Scopus
**Search date:** up to 26 December 2025

****Step 1: #1****
TITLE-ABS-KEY( ("prostate cancer" OR "prostatic cancer" OR "prostate carcinoma" OR "prostatic neoplasm*") AND (prostatectom* OR postprostatectom* OR "post-prostatectomy" OR "radical prostatectomy") AND (salvage OR "salvage radiotherap*" OR "salvage radiation*" OR "postprostatectomy radiotherap*" OR "prostate bed radiotherap*" OR SRT))

****Step 2: #2****
TITLE-ABS-KEY( (PSMA OR "prostate-specific membrane antigen") AND (PET OR "PET/CT" OR "PET MRI" OR "PET/MRI" OR "positron emission" OR "positron emission tomography"))

****Step 3: #3****
TITLE-ABS-KEY((boost* OR "dose escalation" OR escalat* OR intensif* OR "simultaneous integrated boost" OR SIB OR "elective nodal" OR ENRT OR "whole pelvis" OR WPRT OR "pelvic nodal" OR "PET-directed" OR "lesion-directed"))

**Final combination:**
**#1** AND **#2** AND **#3** NOT TITLE-ABS-KEY( "case report" OR "case reports" OR review OR "systematic review" OR "meta-analysis" OR "meta analysis" OR metaanalysis)

#### 4. Embase

**Database:** Embase (via Ovid or Elsevier)
**Search date:** up to 26 December 2025

**Step 1: #1**

(prostate cancer* OR prostatic neoplasm* OR prostate carcinoma* OR prostatic cancer*) AND (prostatectom* OR postprostatectom* OR "post-prostatectomy" OR "radical prostatectomy") AND (salvage OR "salvage therapy" OR "salvage radiotherap*" OR "salvage radiation*" OR "postprostatectomy radiotherap*" OR "prostate bed radiotherap*" OR SRT)

**Step 2: #2**

(PSMA OR "prostate-specific membrane antigen") AND (PET OR "PET/CT" OR "PET MRI" OR "PET/MRI" OR "positron emission" OR "positron emission tomography" )

**Step 3: #3**

(boost* OR "dose escalation" OR escalat* OR intensif* OR "simultaneous integrated boost" OR SIB OR "elective nodal" OR ENRT OR "whole pelvis" OR WPRT OR "pelvic nodal" OR "PET-directed" OR "lesion-directed" )

**Final combination:**

**#1** AND **#2** AND **#3** NOT ("case report" OR "case reports" OR review OR "systematic review" OR
"meta analysis" OR "meta-analysis" OR metaanalysis)

#### 5. Cochrane Library

**Database:** Cochrane Library (CENTRAL and other Cochrane databases)
**Search date:** up to 26 December 2025

**Step 1: #1**
(prostate cancer* OR prostatic cancer* OR prostate carcinoma* OR prostatic neoplasm*) AND (prostatectom* OR postprostatectom* OR "post-prostatectomy" OR "radical prostatectomy") AND (salvage OR "salvage radiotherap*" OR "salvage radiation*" OR "postprostatectomy radiotherap*" OR "prostate bed radiotherap*" OR SRT)

**Step 2: #2**
(PSMA OR "prostate-specific membrane antigen") AND ( PET OR "PET/CT" OR PET-CT OR "PET/MRI" OR PET-MRI OR "positron emission")

**Step 3: #3**
boost* OR "dose escalation" OR escalat* OR intensif* OR "simultaneous integrated boost" OR SIB OR "elective nodal" OR ENRT OR "whole pelvis" OR WPRT OR "pelvic nodal" OR "PET-directed" OR "lesion-directed"

**Final combination:**

**#1** AND **#2** AND **#3** NOT ("case report" OR "case reports" OR review OR "systematic review" OR "meta-analysis" OR metaanalysis)
