# Supplementary material for: PSMA PET–guided intensification of postprostatectomy salvage radiotherapy for prostate cancer: a systematic review and meta-analysis
Source: Front Oncol. 2026 Mar 10;16:1779689. doi: 10.3389/fonc.2026.1779689 (PMC13008707; doi:10.3389/fonc.2026.1779689)
Supplement: Supplementary file 3 [file Table1.docx]

| Supplementary Table S1A. Eligibility and baseline characteristics | | | | | | | | | | | | | | | | | |
| --- | --- | --- | --- | --- | --- | --- | --- | --- | --- | --- | --- | --- | --- | --- | --- | --- | --- |
| Study (year) | Country/Region | Study design | Inclusion criteria | Exclusion criteria | Clinical setting (BCR/persistence; early/delayed SRT) | BCR definition (exact) | PSA persistence definition (exact) | Baseline PSA at PET (median, IQR/range) | Baseline PSA at SRT (median, IQR/range) | PSA kinetics | Pathology: pT | Pathology: pN | Margins (R0/R1) | Gleason/ISUP grade group | Other risk features(EPE/SVI/LVI) | Prior treatments post-RP | Follow-up median (months) |
| Arifin et al., (2023) [14] | Canada (London, ON) | Single-center retrospective cohort study | Men with BF after RP receiving salvage RT (prostate bed and/or pelvic nodes), no disease outside pelvis; PSMA cohort had 18F-DCFPyL PSMA-PET via registry; conventional imaging prior to RT | Excluded: metastatic disease outside pelvis (incl. bone/visceral); prior overlapping pelvic RT; non-standard ADT before surgery; stereotactic RT to pelvic nodes without prostate bed RT; negative PSMA-PET with no salvage RT; non-standard/no dose info; missing data; remained hormone suppressed; MDT/SBRT; duplicate patients; widespread metastases | Post-RP biochemical failure; salvage EBRT; PSMA cohort largely later-salvage (higher PSA) due to PET access limitations | NR (BF/BCR threshold not explicitly stated) | NR | Baseline PSA at PET: NR (PSA at time of PET not separately reported) | Pre-RT PSA median(IQR): 0.48(0.26–0.73) vs 0.20(0.14–0.28); matched 0.34(0.22–0.60) vs 0.21(0.15–0.38) | NR | NR | LN involvement: 6/44 (13.6%) vs 4/80 (5.0%); matched 5/34 (14.7%) vs 4/34 (11.8%) | Positive margins: 20/44 (45.5%) vs 44/80 (55.0%); matched 16/34 (47.1%) vs 20/34 (58.8%) | Gleason grade group distribution reported (GG2 most common; GG3–5 present; matched similar) | EPE: 26/44 (59.1%) vs 54/80 (67.5%); SVI: 10/44 (22.7%) vs 21/80 (26.3%); CAPRA-S median(IQR) 5(4–7) vs 5(4–6) | Prior post-RP: excluded previous pelvic RT overlap; excluded non-standard ADT before surgery; ADT during salvage allowed (physician discretion); conventional restaging with CT/bone scan | Matched median 26 months (IQR 18.8–33) |
| Petit et al ., (2025) [7] | Canada | Bi-institutional prospective observational cohort study | Planned salvage RT post-RP with BCR (PSA >0.1 ng/mL); ECOG 0–1; Charlson Comorbidity Index <5 | Exclusions included androgen deprivation therapy within 12 months and prior PSMA-PET (per CONSORT) | BCR after RP; salvage RT (delayed: median ~19.5–19.9 mo from surgery to enrollment) | Eligibility BCR: PSA >0.1 ng/mL | NR | 0.3 ng/mL (0.1–2.4) (experimental; enrollment/PET baseline) | NR (PSA at SRT not separately reported; control enrollment PSA 0.3 [0.1–3.0]) | NR | pT2 45.3% / pT3–4 54.7% (experimental); pT2 37.5% / pT3–4 62.5% (control) | N0 87.5% / N1 10.9% / Nx 1.6% (experimental); N0 90.6% / N1 7.8% / Nx 1.6% (control) | Positive margins: 53.1% (experimental) vs 31.5% (control) | Grade group (1–5): 17.2/20.3/43.8/14.1/4.7% (experimental) vs 28.1/26.6/32.8/7.8/4.7% (control) | NR (EPE/SVI/LVI etc not detailed in main text/table) | Hormone therapy at baseline: 54/64 (84%) vs 55/64 (86%); time since surgery median 19.5 vs 19.9 months (range 1.0–188 vs 1.4–130.8); exclusions included ADT within 12 mo | 37 (range 7–60) months |
| Bluemel et al., (2016) [15] | Germany | Bi-institutional retrospective cohort study | Post-RP prostate cancer with persisting PSA (n=16) or BCR (n=29) scheduled for prostate-bed SRT; hormone-naïve at imaging; underwent 68Ga-PSMA PET/CT before SRT decision | Not explicitly reported | PSA persistence or BCR after RP; SRT intended for prostate bed (timing early vs delayed NR; time since RP mean 5.7 y) | NR | NR | 0.67 ng/mL (range 0.10–11.22; mean 1.30±2.06) | NR overall (subset treated+FU n=21: median 0.60; range 0.10–3.51) | PSADT mean 7.4±8.0 mo (range 0.0–31.5; median 4.8) | Pathologic stage distribution: T2 ~25/45; T3/ypT3 ~20/45 | N0 33/45; N1 11/45; Nx 1/45 | R0 30/45; R1 14/45; Rx 1/45 | Gleason mean 7±1 (range 4–9) | D’Amico: high 39/45; intermediate 4/45; low 2/45 | Hormone-naïve at PET; no ADT before imaging; no patient received ADT during SRT (prior RT NR) | Median 6.92 mo (range 1.15–24.36) among n=21 with follow-up |
| Dhere et al., (2025) [16] | USA | Single-center prospective observational cohort study | Detectable PSA following radical prostatectomy; no prior pelvic radiotherapy; no extra-pelvic disease on 99mTc-MDP bone scan and CT/MRI A/P; underwent CT simulation then PET/CT per randomization | Extra-pelvic uptake on PET/CT leading to off-study management; withdrawal/declined RT; (patients not receiving RT on trial excluded from analysis) | Post-RP detectable PSA (BCR/PSA persistence not explicitly separated); early salvage common (discussion notes many treated before PSA 0.2 ng/mL) | NR (trial used 'detectable PSA following prostatectomy'; no explicit BCR threshold/confirmation described) | NR | NR (PET-time PSA not separately reported); pre-radiation PSA medians: Fluciclovine 0.27 (0.14–0.74), PSMA 0.35 (0.18–0.74) ng/mL | Pre-radiation PSA: Fluciclovine 0.27 (0.14–0.74; 0.02–19.4), PSMA 0.35 (0.18–0.74; 0.03–75.9) ng/mL | NR | NR | Pathologic nodal status (enrolled): N+ 12/70 (17.1%) fluciclovine; 16/70 (22.8%) PSMA | Margin+ (enrolled): 45/70 (64.2%) fluciclovine; 44/70 (62.8%) PSMA | Grade group (enrolled): ≤3 48/70 (68.6%) vs 40/70 (57.2%); ≥4 22/70 (31.4%) vs 30/70 (42.8%) (fluciclovine vs PSMA) | ECE (enrolled): 24/70 (34.2%) vs 23/70 (32.8%); SVI: 24/70 (34.2%) vs 20/70 (28.6%) (fluciclovine vs PSMA) | No prior pelvic RT; ADT intent at enrollment ~60% both arms (fluciclovine 42/70; PSMA 43/70); other prior tx NR | NR (acute toxicity assessed weekly during RT and at first post-RT visit ≤90 days) |
| Gunnlaugsson et al., (2022) [17] | Sweden | Single-center prospective phase II observational cohort study | Post-RP; pN0 or pNx; M0; age ≥18; confirmatory PSA ≥0.15 ng/mL | Metastatic disease (M1); nodal involvement at RP (N1); previous hormone therapy; previous pelvic radiotherapy | BCR after RP; early salvage (median PSA at start SRT 0.25 ng/mL); median RP→SRT 38 months | Pre-SRT: confirmatory PSA ≥0.15 ng/mL; Post-SRT BCR used in FFS: PSA rise ≥0.2 ng/mL above nadir confirmed by another PSA | NR | NR (PET performed before RT but PSA at PET not explicitly reported) | 0.25 ng/mL (range 0.15–0.7); responders 0.23 (0.15–0.6); non-responders 0.26 (0.15–0.7) | NR (no PSADT/velocity reported) | NR | Eligibility: pN0/pNx (no breakdown) | NR | NR | NR | No previous hormone therapy; no previous pelvic radiotherapy | 38 months (range 7–62) |
| Janbain et al., (2024) [18] | Germany; Cyprus; Australia; Italy; Switzerland | Multicenter retrospective cohort study | Open or laparoscopic RP; PSMA-PET–based sRT for PSA persistence/recurrence; PSA ≥0.1 ng/mL postprostatectomy | Distant metastases on PSMA-PET or CT; ADT initiated before imaging; insufficient clinical data; no prostatic fossa in sRT field; PSMA-PET+ lesions outside sRT field | Post-RP PSA persistence or recurrence; early sRT common (PSA ≤0.5 ng/mL: 61.3% in learning cohort; 61.3% approx) | BR after sRT: 2 consecutive rising PSA values >0.2 ng/mL | NR (variable recorded: PSA persistence after surgery yes/no) | NR | NR (PSA before sRT categories: 0.01–0.2 23.9%; >0.2–0.5 37.4%; >0.5–1 16.7%; >1 22.0%) | NR | pT2 44.7%; pT3a 31.8%; pT3b 22.8%; pT4 0.7% | NR | R0 65.4%; R1 31.8% (R2 0.3%; Rx 2.5%) | ISUP 1+2 36.1%; 3 31.5%; 4 15.2%; 5 17.3% | NR | Excluded if ADT started before PSMA-PET/CT; other prior treatments/previous RT NR | NR |
| Jani et al., (2025) [19] | USA | Single-center retrospective cohort study | Detectable PSA after radical prostatectomy AND no metastases on conventional imaging; consented participants; ADT before randomization allowed if PSA detectable at enrollment | Ineligibility criteria stated as identical to EMPIRE-1 (not fully detailed in text); metastases on conventional imaging not eligible | Post-RP biochemical progression with detectable PSA; conventional imaging negative; salvage/curative-intent RT; early/delayed SRT not explicitly separated | Eligibility BCR threshold NR (described as 'detectable PSA/biochemical progression after prostatectomy'); outcome event includes PSA >0.2 ng/mL from nadir followed by another rise | NR (PSA persistence definition not explicitly stated); outcome event includes 'persistent PSA' | PET-time PSA NR; closest reported is pre-RT PSA: Arm 1 0.2 (0.2–0.8), range 0.01–19.4; Arm 2 0.4 (0.2–0.8), range 0.03–75.9 ng/mL | Pre-RT PSA: Arm 1 0.2 (0.2–0.8), 0.01–19.4; Arm 2 0.4 (0.2–0.8), 0.03–75.9 ng/mL | NR | pT3: Arm 1 48/70 (69%); Arm 2 50/70 (71%) | pN+: Arm 1 12/70 (17%); Arm 2 16/70 (23%) | Positive surgical margin: Arm 1 45/70 (64%); Arm 2 44/70 (63%) | Gleason score 8: Arm 1 22/70 (31%); Arm 2 30/70 (43%) (grade group NR) | SVI: Arm 1 24/70 (34%); Arm 2 20/70 (29%) | Post-RP treatments: ADT intent at baseline common; ADT before randomization discouraged but allowed; prior RT NR (criteria per EMPIRE-1) | 31.2 (IQR 21.6–48.0) |
| Kirste et al., (2021) [20] | Germany; Switzerland | Multicenter retrospective cohort study | Prior radical prostatectomy; no distant metastases at initial diagnosis; oligo-recurrent PC with 68Ga-PSMA-PET/CT–positive local (prostate bed) and/or nodal (N1) and/or distant metastases; curative-intent RT to all PSMA-ligand positive lesions; any PSA at time of PSMA PET accepted; max 5 visceral and/or bone metastases | Recurrences under active ADT; previous chemotherapy for PC; history of previous RT of prostate bed and/or pelvic lymph nodes after earlier biochemical recurrence following RP | Biochemical recurrence after RP with PSMA PET–positive oligo-recurrence (local/nodal/metastatic); salvage/MDT setting; early vs delayed SRT NR | NR for inclusion (biochemical recurrence per local practice; any PSA accepted) | NR | PSA at start of RT/MDT: 1.2 ng/mL (range 0.04–47.5); same time as pre-RT PSMA PET (exact PET-time PSA NR) | 1.2 ng/mL (range 0.04–47.5) | PSADT/velocity NR; 2 mo after RT PSA decreased in 364/394 (92.4%); median nadir 0.07 ng/mL (range 0.01–13.71) | pT1c 8 (2.0%); pT2a 15 (3.8%); pT2b 11 (2.8%); pT2c 126 (32.0%); pT3a 90 (22.8%); pT3b 134 (34.0%); pT4 9 (2.3%); Tx 1 (0.3%) | pN0 261 (66.2%); pN1 120 (30.5%); Nx 13 (3.3%) | R0 217 (55.3%); R1/R2 166 (42.4%); Rx 11 (2.3%) | Gleason 6: 21 (5.3%); 7a: 82 (20.9%); 7b: 127 (32.2%); 8: 51 (12.9%); 9: 108 (27.4%); 10: 3 (0.8%); unknown: 2 (0.5%) | D’Amico high/very high risk 379/394 (96.1%); Gleason ≥8: 162/394 (41.1%); LN+ disease at diagnosis 120/394 (30.5%); other features (EPE/SVI/LVI) NR | Active ADT at recurrence excluded; prior chemo excluded; prior prostate bed/pelvic RT after earlier BCR excluded (no re-irradiation); additive ADT during oligometastatic RT allowed (see ADT use) | 28 months (range 1–71) |
| Rogowski et al., (2022) [21] | Germany | Single-center retrospective cohort study | Histologically confirmed PCa; post-RP with persistent or rising PSA (bcP/bcR); PSMA PET/CT performed; PSMA PET/CT–based sENRT for LN recurrence; allowed M1a lumboaortic LN below renal arteries | No prior RT to prostate/prostate bed; excluded M1b/M1c disease | bcP 76%; bcR 24%; nodal recurrence; timing early/delayed NR (median RP→bcR 22.5 months) | NR (study uses term bcR; exact PSA threshold not specified) | NR (described as persistent or rising PSA after RP) | Overall 1.4 (0.1–40.1) ng/mL; bcP 1.7 (0.1–40.1); bcR 0.6 (0.3–5.1) | NR (analyzed as PSA <1 vs ≥1 ng/mL before sRT) | NR | pT2a 1%; pT2c 20%; pT3a 25%; pT3b 51%; pT4 3% | pN0 54%; pN1 42%; pNx/cN0 2%; unknown 2% | Positive surgical margins 52% | ISUP 1:2%; 2:12%; 3:25%; 4:18%; 5:43% | NR (only pT/pN/ISUP/margins reported) | No prior RT to prostate/prostate bed (re-irradiation excluded); other prior systemic tx NR |  |
| Schmidt-Hegemann et al., (2019) [22] | Germany | Bi-institutional retrospective cohort study | Post-RP biochemical recurrence; PSMA PET/CT performed before salvage RT; no prior salvage treatment beforehand; received salvage RT (prostatic fossa ± pelvic LNs) with PET-guided dose escalation/field enlargement as indicated | PSA persistence excluded; distant metastases on PSMA PET/CT excluded | BCR after RP (PSA persistence excluded); early salvage common (median PSA ~0.44) | NR (biochemical relapse after RP; exact PSA trigger not specified) | PSA persistence excluded (definition NR) | 0.43 ng/mL (range 0.10–6.24) at PSMA PET/CT; PET+ 0.78 (0.23–6.24), PET− 0.30 (0.10–3.24) | 0.44 ng/mL (range 0.11–6.24) before RT; PET+ 0.68 (0.30–6.24), PET− 0.34 (0.11–3.24) | NR | pT2a/b 11%; pT2c 49%; pT3a 26%; pT3b 12%; pT4 2% | pN0 73%; pN1 12%; pNx/cN0 15% | Positive surgical margins 31% | Gleason: 6 9%; 7a 27%; 7b 37%; 8 17%; 9 10% | NR | No salvage treatment beforehand; ADT started mainly in PET-positive patients (25/42) before RT; 1 PET-negative also received ADT; no prior RT reported | 23 mo (range 1–47) |
| Spohn et al., (2022) [23] | Germany; Italy (Bologna) | Multicenter retrospective cohort study | Post-RP; PSA persistence (PSA≥0.1) or recurrence (PSA≥0.2); 68Ga-PSMA11 PET prior to sRT; PET-positive LR and/or pelvic NR; treated with PSMA PET-guided sRT | Excluded: distant mets (LN above iliac bifurcation, bone, visceral) on PET; ADT prior to PET; equivocal/single-slice PET findings not suitable for contouring | PSA persistence and/or BCR after RP; PSMA PET performed pre-sRT; early vs delayed sRT NR | Recurrence: PSA ≥0.2 ng/mL as nadir after surgery | PSA persistence: PSA after surgery ≥0.1 ng/mL | NR | NR (reported as categories pre-sRT PSA: <0.5 28%; ≥0.5 70%) | NR | pT2a–c 75/235 (32%); pT3–4 134/235 (57%); n/a 26/235 (11%) | pN0 130/235 (55%); pN1 51/235 (22%); n/a 54/235 (23%) | R0 98/235 (42%); R1 73/235 (31%); n/a 64/235 (27%) | ISUP 1–2: 45/235 (19%); ISUP 3–5: 185/235 (79%); n/a 5/235 (2%) | NR (EPE/SVI/LVI not reported) | Excluded if ADT before PSMA PET; prior RT/re-irradiation info NR | 24 months (IQR 16–41) |
| Tamihardja et al., (2022) [24] | Germany | Single-center retrospective cohort study | Post-RP biochemical relapse referred for SRT; pre-SRT PSMA PET/CT; PSMA PET+ macroscopic prostatic fossa recurrence (± locoregional LN) treated with dose-escalated SRT | Excluded PET-negative; distant metastasis treated palliatively; nodal-only recurrence treated with nodal SRT (reported elsewhere); other exclusions/previous RT NR | BCR setting (median ~68 months from RP to SRT); PSA persistence not described | NR (biochemical relapse after RP; threshold/confirmation not specified) | NR | NR (PSA at PET not separately reported) | 0.8 ng/mL (IQR 0.4–1.7) | NR | pT2b 1.7%; pT2c 49.2%; pT3a 23.7%; pT3b 22.0%; N/A 3.4% | pN0 88.1%; pN1 8.5%; N/A 3.4% | R0 61.0%; R1 27.1%; N/A 11.9% | ISUP group 1 25.4%; group 2–3 45.8%; group 4–5 27.1%; N/A 1.7% | D’Amico high risk 96.6%; other features (EPE/SVI/LVI) NR | Prior post-op RT/ADT NR; concomitant ADT during SRT given in 32.2% (recommended if GS≥8 or pre-SRT PSA≥0.7 per German guidelines) | 38.2 months (IQR 29.0–48.3) |
| Trapp et al., (2024) [25] | Germany/Switzerland/Australia/Cyprus/Italy | Multicenter retrospective cohort study | Post-RP patients with PSMA-positive nodal recurrence on PSMA PET/CT treated with elective pelvic nodal RT (WPRT or HPRT) as salvage RT for PSA recurrence or PSA persistence | Excluded incomplete/insufficient follow-up; excluded patients not receiving RT of lymphatic pathways (e.g., SBRT-only); dataset cleaning from 1222 PSMA-PET/CT-guided salvage RT cases to 273 pelvic nodal RT cases | Nodal recurrence after RP; PSA recurrence and PSA persistence included (proportions differed after matching) | NR (BCR definition after RP not specified) | NR (PSA persistence definition not specified) | Reported as categories (PSA before PET/CT): ≤0.2: 1/51 (2%) vs 1/51 (2%); 0.21–0.5: 11 (22%) vs 12 (24%); 0.51–1.0: 13 (26%) vs 9 (18%); >1.0: 26 (51%) vs 29 (57%) | NR | NR | pT2 21 (41%) vs 27 (53%); pT3a 9 (18%) vs 11 (22%); pT3b 16 (31%) vs 10 (20%); pT4 1 (2%) vs 0; missing 8% vs 6% (WPRT vs HPRT) | pN0 34 (67%) vs 37 (73%); pN1 11 (22%) vs 6 (12%); missing 12% vs 16% | R0 27 (53%) vs 37 (73%); R1 15 (29%) vs 8 (16%); missing 18% vs 12% | ISUP 1–2: 15 (29%) vs 17 (33%); ISUP 3: 11 (22%) vs 14 (28%); ISUP 4: 15 (29%) vs 11 (22%); ISUP 5: 10 (20%) vs 9 (18%) | NR | NR (prior treatments not detailed; SBRT-only cases excluded) | 29 overall; 40 (14–84) WPRT vs 28 (12–64) HPRT |
| Fuertes Vallés et al., (2025) [26] | Spain | Single-center retrospective cohort study | Post-RP patients with isolated prostate bed relapse (IPBR) detected on PSMA PET/CT and mpMRI; lesion visible on TRUS and technically implantable | Prior prostate-bed irradiation; nodal or distant relapse; multicentric lesions; endoluminal lesions; life expectancy <5y | BCR setting with macroscopic IPBR; salvage/curative intent (timing relative to RP variable; median 42 months to failure) | NR | NR (not a PSA persistence cohort) | 0.41 ng/mL (range 0.20–1.06) [before brachytherapy / PSMA imaging] | 0.41 ng/mL (range 0.20–1.06) [before HDR; EBRT baseline NR] | PSA doubling time: median 10.2 months (range 3.0–38.0) | T1–2a 6.3%; T2b–2c 62.5%; T3a 31.3% | NR | Positive margins 50% (8/16); R0/R1 breakdown otherwise NR | ISUP GG1 31.3% (5/16); GG2 56.3% (9/16); GG3 6.3% (1/16); GG4 6.3% (1/16) | SVI 0%; perineural involvement 87.5% (14/16); extracapsular spread 37.5% (6/16) | No prior prostate-bed RT (excluded); other post-RP tx NR | 44.4 (range 27.6–62.4) |
| Vogel et al., (2021) [27] | Germany | Single-center retrospective cohort study | Post-RP PCa with PSA nadir <0.1 ng/mL and biochemical relapse; salvage RT to prostate bed (PB) ± elective pelvic LNs; DE-SRT arm received PSMA PET–guided dose escalation (SIB) to PET-positive lesion(s) | Excluded: distant metastases; 3D-CRT; choline PET; sequential boost (non-SIB); PET-positive patients without dose escalation; PB EQD2 <66 Gy | BCR after RP (PSA nadir <0.1 ng/mL); PSA persistence NR; early/delayed SRT NR | NR (biochemical relapse after PSA nadir <0.1 ng/mL; subgroup analysis uses PSA at recurrence >0.2 ng/mL) | NR | Median PSA at recurrence (proxy PSA at PET): overall 0.32 (0.02–22.00); C-SRT 0.21 (0.02–5.64); DE-SRT 0.45 (0.02–22.00) | Median PSA before RT: overall 0.44 (0.02–16.02); C-SRT 0.33 (0.02–16.02); DE-SRT 0.52 (0.02–16.02) | NR | pT1c 1 (0.5%): 1,0; pT2 5 (2.5%): 2,3; pT2a 10 (5.1%): 3,7; pT2b 5 (2.5%): 3,2; pT2c 78 (39.2%): 40,38; pT3 2 (1.0%): 1,1; pT3a 52 (26.1%): 28,24; pT3b 41 (20.6%): 19,22; pT4 2 (1.0%): 1,1; missing 3 (1.5%): 0,3 | pN0 165 (82.9%): 84,81; pN1 26 (13.1%): 12,14; pNx 6 (3.0%): 2,4; missing 2 (1.0%): 0,2 | R0 142 (71.4%): 71,71; R1 45 (22.6%): 26,19; Rx 7 (3.5%): 1,6; missing 5 (2.5%): 0,5 | ISUP/GG: 1=12 (6.0%): 9,3; 2=80 (40.2%): 41,39; 3=52 (26.1%): 20,32; 4=19 (9.5%): 12,7; 5=30 (15.1%): 14,16; GS7-unspecified=2 (1.0%): 2,0; missing=4 (2.0%): 0,4 | ECE/SVI NR | Additive ADT 40/199 (20.1%): 12,28; distant mets excluded | Median follow-up 13.6 months (0.4–70.0); C-SRT 18.9 (0.4–70.0); DE-SRT 10.7 (0.7–59.4) |
| ENRT, elective nodal radiotherapy; LN, lymph node; LND, lymph node dissection; LVI, lymphovascular invasion; MDT, metastasis-directed therapy; miTNM, molecular imaging TNM; NR, not reported; pN, pathological nodal stage; pT, pathological tumor stage; PRO, patient-reported outcome; QoL, quality of life; ¹⁸F, fluorine-18; ⁶⁸Ga, gallium-68. | | | | | | | | | | | | | | | | | |
