# Supplementary material for: PSMA PET–guided intensification of postprostatectomy salvage radiotherapy for prostate cancer: a systematic review and meta-analysis
Source: Front Oncol. 2026 Mar 10;16:1779689. doi: 10.3389/fonc.2026.1779689 (PMC13008707; doi:10.3389/fonc.2026.1779689)
Supplement: Supplementary file 4 [file Table2.docx]

| Supplementary Table S1B. Imaging protocol and findings | | | | | | | | | | | | |
| --- | --- | --- | --- | --- | --- | --- | --- | --- | --- | --- | --- | --- |
| Study (year) | PSMA PET modality (PET/CT vs PET/MRI) | Tracer | Imaging protocol | Positivity criteria | miTNM reported | PET detection rate (%) | PET pattern: local bed (%) | PET pattern: pelvic nodes (%) | PET pattern: extra-pelvic nodes (%) | PET pattern: bone (%) | PET pattern: visceral/other (%) | Number of lesions / nodes |
| Arifin et al., (2023) [14] | PSMA-PET/CT: NR (reported as PSMA-PET; modality details not specified in this paper) | 18F-DCFPyL | NR (stated “described in previous publication”) | NR | No | 40.9% (18/44 had PET-positive findings within pelvis) | 4.6% (2/44) | 36.4% (16/44) | 0% reported | 0% reported | 0% reported | NR |
| Petit et al ., (2025) [7] | PSMA-PET/CT | NR | NR (protocol details not in main text; PET interpreted by 2 nuclear medicine physicians) | Lesions rated ‘probable’ or ‘definite’ by 2 board-certified nuclear medicine physicians; ‘probable/definite’ lesions used for RT intensification | NR | 33/64 (52%) had new lesions on PSMA-PET/CT prompting intensification | NR | NR | NR | NR | NR | NR |
| Bluemel et al., (2016) [15] | PET/CT | 68Ga-PSMA I&T (EuK-Sub-kf(3-iodo-y)-68Ga-DOTAGA) | Injected activity 141±19 MBq (97–184); furosemide 10–20 mg; uptake time 60 min; skull base→prox thighs 2–3 min/bed; contrast CT in 25/45 | PET+: focal non-physiologic uptake higher than background; CT+: morphologic malignancy criteria (e.g., LN short axis ≥10 mm, osteoblastic bone lesions) | No | 24/45 (53.3%) | 11/45 (24.4%) local recurrence | ~11/45 (24.4%) with pelvic LN involvement (includes LNM ± local/bone) | At least 2/45 (4.4%) with retroperitoneal LN involvement (exact total NR) | 2/45 (4.4%) bone metastases | 2/45 (4.4%) rectal soft-tissue lesions | NR (oligometastatic defined as ≤5 lesions in ≤3 organs; >5 metastases in 2 patients) |
| Dhere et al., (2025) [16] | PET/CT | 68Ga-PSMA-11 (Arm 2); comparator tracer 18F-fluciclovine (Arm 1) | NR (detailed injected activity/uptake time/reconstruction not provided in manuscript text) | Iso-SUV volumes based on prior positivity criteria and clinical factors; PET fused via deformable registration to CT sim | No | NR (detection/positivity rates not primary endpoint in this analysis) | NR | NR | NR | NR | NR | NR |
| Gunnlaugsson et al., (2022) [17] | PET/CT | 68Ga-PSMA-11 | Injected 2.5 MBq/kg (max 300 MBq); scan ~1 h post-injection; mid-thigh to skull base; GE Discovery 690 | Any uptake not typical physiologic distribution regarded suspicious; 2 experienced readers | NR/No | 26% overall (25/97); responders 9% (3/34); non-responders 35% (22/63) | 9% (9/97) | 10% (10/97) | 0% / NR | 5% (5/97) | 1% (bone+liver 1/97) | NR (protocol excluded >3 LN mets but lesion counts not reported) |
| Janbain et al., (2024) [18] | PSMA-PET (PET/CT vs PET/MRI NR; CT referenced) | PSMA ligands (NR specific tracer) | NR | NR | NR | NR (local recurrence 42.5%; pelvic nodes 30.4%; distant mets excluded) | 42.5% (437/1029) | 30.4% (313/1029) | Excluded (distant mets excluded at baseline) | Excluded (distant mets excluded at baseline) | Excluded (distant mets excluded at baseline) | NR |
| Jani et al., (2025) [19] | PET/CT | Arm 2: [68Ga]-PSMA-11; Arm 1: [18F]-fluciclovine | NR (protocol details referenced; not fully specified in text) | NR (protocol referenced) | NR | Detection (any uptake) in PET subset: Arm 1 62/65 (95.4%); Arm 2 42/69 (60.9%) | Local/prostate bed only uptake: Arm 1 47/65 (72%); Arm 2 24/69 (35%) | Pelvic nodes ± prostate bed uptake: Arm 1 10/65 (15%); Arm 2 10/69 (15%) | NR (not separated); reported combined extraplelvic uptake: Arm 1 5/65 (7.7%); Arm 2 8/69 (12%) | NR (not separated); reported combined extraplelvic uptake: Arm 1 5/65 (7.7%); Arm 2 8/69 (12%) | NR (not separated); reported combined extraplelvic uptake: Arm 1 5/65 (7.7%); Arm 2 8/69 (12%) | NR |
| Kirste et al., (2021) [20] | PET/CT (contrast-enhanced or low-dose CT) | 68Ga-PSMA-11 | Imaging ~1 h after IV tracer; furosemide IV 30 min prior; void before scan; EANM/SNMMI guideline-based; PET window settings example SUV 0–10; injected activity/reconstruction NR | Visual definition: focal tracer accumulation greater than normal/physiological local background activity | No (reported N/M stage at recurrence, not miTNM explicitly) | 100% (PSMA-ligand positive lesions required for inclusion) | 29.4% (116/394 had PSMA+ prostate bed recurrence) | 53.6% (211/394 had PSMA+ lymph node recurrence, N1) | 14.3% (M1a: 57/394) | 18.1% (M1b: 72/394) | 1.8% (M1c: 7/394) | NR (inclusion allowed max 5 visceral and/or bone metastases) |
| Rogowski et al., (2022) [21] | PET/CT | 68Ga-PSMA-11 75%; 18F-PSMA-1007 25% | PET/CT ~60 min post-injection; furosemide 20 mg at injection if no contraindication; scanners: Siemens Biograph 64 or GE Discovery 690; contrast-enhanced diagnostic CT or low-dose CT | Visual PSMA uptake |  |  |  |  |  |  |  |  |
| Schmidt-Hegemann et al., (2019) [22] | PET/CT | 68Ga-PSMA (PSMA-HBED-CC) | Mean 205 MBq; imaging 60 min post-injection; furosemide 20 mg at injection if no contraindication; diagnostic contrast CT or low-dose CT | Visual uptake above background and non-physiologic uptake | No | 47% PET-positive (42/90) | Any fossa involvement 33% (30/90); fossa only 27% (24/90) | Any pelvic nodes 20% (18/90); pelvic LN only 13% (12/90) | 0% (distant/nonregional LN mets excluded) | 0% (bone mets excluded) | 0 | NR |
| Spohn et al., (2022) [23] | PET/CT | 68Ga-PSMA-11 | ~60 min uptake; ~1.8–2.2 MBq/kg; CT slice thickness 2 mm; scanners EARL1-accredited (EANM compliant) | Focal uptake > adjacent background in >1 slice considered PCa; equivocal/one-slice findings not segmented | NR | 100% (only PET-positive included) | Local bed/LR: 140/235 (60%) | Pelvic nodes/NR: 138/235 (59%) | 0% (excluded LN above iliac bifurcation) | 0% (bone mets excluded) | 0% (visceral mets excluded) | NR |
| Tamihardja et al., (2022) [24] | PSMA PET/CT | 68Ga-PSMA I&T (74.6%); 18F-PSMA-1007 (25.4%) | NR (injection activity/uptake time/reconstruction not reported in extracted text) | Macroscopic local recurrence defined as relapse visible on PSMA PET/CT | NR | Not applicable (selected PSMA PET+ cohort); among 111 staged: 81.1% PET+; 53.2% had PET+ fossa recurrence | 100% (by inclusion); fossa only 81.4% | 18.6% (11/59) locoregional LN+ | 0%/NR (extra-pelvic nodes not reported in analyzed cohort; distant mets excluded) | 0%/NR (bone mets not reported in analyzed cohort; distant mets excluded) | 0%/NR (visceral/other not reported in analyzed cohort; distant mets excluded) | LN metastases count: 0 (81.4%); 1 (6.8%); 2 (5.1%); 3 (3.4%); ≥4 (3.4%) |
| Trapp et al., (2024) [25] | PSMA PET/CT | 68Ga-PSMA or 18F-PSMA | NR (local practice; details referenced to prior publication) | NR | No/NR | NR | Local recurrence on PET/CT: 17/51 (33%) vs 3/51 (6%) | PSMA-positive pelvic nodal recurrence by inclusion (pelvic RT of lymphatic pathways) | NR | NR | NR | NR (dataset did not include number/pattern of LNM) |
| Fuertes Vallés et al., (2025) [26] | PSMA PET/CT (plus mpMRI; TRUS-visible lesion required) | NR | NR | GTV defined as PSMA/MRI-positive lesion visible on TRUS; eligibility required IPBR on PSMA PET/CT and mpMRI | No/NR | 100% (eligibility required PSMA-detected IPBR) | 1 | 0% (excluded if nodal relapse) | 0% (excluded) | 0% (excluded) | 0% (excluded) | Single local lesion (IPBR) per inclusion; exact counts NR |
| Vogel et al., (2021) [27] | PSMA PET/CT or PET/MRI (per EANM/SNMMI guidelines) | [68Ga]PSMA-11; [18F]rhPSMA-7; [18F]rhPSMA-7.3; [18F]PSMA-1007; No PET in 55/98 C-SRT | Pre-RT (used for planning) | NR | No | NR | Local bed involvement among DE-SRT: 83/101 (82.2%) [local-only 58 + both 25] | Pelvic LN involvement among DE-SRT: 43/101 (42.6%) [LN-only 18 + both 25] | Extra-pelvic nodes: NR (distant metastases excluded) | 0 (distant metastases excluded) | 0 (distant metastases excluded) | NR |
| ENRT, elective nodal radiotherapy; LN, lymph node; LND, lymph node dissection; LVI, lymphovascular invasion; MDT, metastasis-directed therapy; miTNM, molecular imaging TNM; NR, not reported; pN, pathological nodal stage; pT, pathological tumor stage; PRO, patient-reported outcome; QoL, quality of life; ¹⁸F, fluorine-18; ⁶⁸Ga, gallium-68. | | | | | | | | | | | | |
