# Supplementary material for: PSMA PET–guided intensification of postprostatectomy salvage radiotherapy for prostate cancer: a systematic review and meta-analysis
Source: Front Oncol. 2026 Mar 10;16:1779689. doi: 10.3389/fonc.2026.1779689 (PMC13008707; doi:10.3389/fonc.2026.1779689)
Supplement: Supplementary file 5 [file Table3.docx]

| Supplementary Table S1C. Radiotherapy and systemic treatment details | | | | | | | | | | | | | | | |
| --- | --- | --- | --- | --- | --- | --- | --- | --- | --- | --- | --- | --- | --- | --- | --- |
| Study (year) | Intensification type classification(Boost/SIB; ENRT/WPRT; MDT; combo) | RT intent | RT fields (detailed) | Target definition guideline | Technique (IMRT/VMAT/protons/SBRT) | IGRT (Y/N; method) | Prostate bed dose (Gy/fx) | Pelvic elective dose (Gy/fx) | Node/lesion boost dose (Gy/fx; SIB/SABR) | SBRT dose if applicable (Gy/fx) | OAR constraints / planning notes | ADT use (n, %) | ADT regimen(LHRH/antiandrogen/ARPI) | ADT duration (months) | Other systemic therapy |
| Arifin et al., (2023) [14] | Combo: ENRT/WPRT (pelvic nodal irradiation) ± SIB boost to PSMA-avid nodes/bed nodule | Salvage intent; MDT/SBRT excluded | Prostate bed only vs prostate bed + pelvic nodes; in PSMA cohort: more pelvic node coverage and occasional SIB boost | NR | VMAT | NR | 66 Gy / 33 fx (prostate bed) | 50.4 Gy / 33 fx (pelvic elective) | 60–70 Gy / 33 fx (SIB/in-field boost to PSMA-avid target) | NR | NR | ADT use: 16/44 (36.4%) vs 18/80 (22.5%); matched 10/34 (29.4%) vs 10/34 (29.4%) | NR | Median 6 months (IQR 4.67–6.05) in PSMA vs 6.03 (5.95–11.99) in control; range ~4 months–2 years | NR |
| Petit et al ., (2025) [7] | LN boost (aim EQD2 66 Gy): 19 (30%); prostate bed boost (aim EQD2 77 Gy): 15 (23%); metastasis-directed RT (aim EQD2 66 Gy): 2 (3%); overlap possible | Salvage intent; allowed PET-directed boost and metastasis-directed RT when indicated | Prostate bed for all; elective pelvic nodes per risk factors; PET-positive sites boosted (nodes/bed) and/or treated with MDT RT | NR | NR (IMRT/VMAT/protons not specified) | NR | Prostate bed: 66 Gy/33 fx (94% experimental; 97% control) or 70 Gy/35 fx (6% experimental; 3% control) | Pelvic elective (if used): 44 Gy/22 fx most common (77% both arms); other regimens 42–48 Gy in 20–25 fx (see Table 1) | Boost targets: pelvic LN boost aim EQD2 66 Gy; prostate bed boost aim EQD2 77 Gy (fractionation NR) | MDT RT aim EQD2 66 Gy (fractionation/SBRT details NR) | NR | 54/64 (84%) vs 55/64 (86%) | NR | NR | NR |
| Bluemel et al., (2016) [15] | PET-guided boost/dose escalation + ENRT/WPRT (pelvic±retroperitoneal) ± MDT (bone); ADT when >5 mets | Salvage/curative-intent SRT; MDT for oligometastatic disease | Prostate bed ± pelvic nodes (external/internal iliac/obturator to promontory) ± retroperitoneal (to renal vessels); PET+ lesion boosts; bone lesions; rectal wall when indicated | Prostate-bed CTV per Poortmans et al.; nodal CTV defined as 5-mm margin around vessels | IG-IMRT (6-MV photons) | Yes; daily CBCT | 66–70 Gy (SIB + sequential); morphologic local recurrence up to 76 Gy | 50.4 Gy (1.8 Gy/fx) | LN: SIB 56 Gy (2 Gy/fx) + sequential boost 60–66 Gy; bone lesions 66 Gy | NR | Margins: PTV bed 10 mm (PTV SIB 5 mm); PTV LN 5 mm; PTV SIB LN 10 mm around tracer uptake | 0% at imaging (hormone-naïve); ADT recommended/started in 2/38 (5.6%) who followed panel | NR | NR | NR |
| Dhere et al., (2025) [16] | Boost/SIB to PET uptake within prostate bed (70.2–76.0 Gy) and/or pelvic nodes/upper pelvis (54–56 Gy) (PET-directed dose escalation) | Post-prostatectomy salvage RT (curative intent) | Prostate bed alone for pN0 with no pelvic nodal uptake; prostate bed + pelvic nodes for pN+ and/or pelvic nodal PET uptake | CTVPB per RTOG post-prostatectomy consensus; CTVPLV per RTOG pelvic node atlas | VMAT (Eclipse planning) | NR (IGRT not specified; CT simulation full bladder/empty rectum; PTV coverage ≥95%) | PTVPB 64.8–70.2 Gy in 1.8 Gy/fx | PTVPLV 45–50.4 Gy in 1.8 Gy/fx (when treated; Table 2 subsets: 15 fluciclovine and 17 PSMA) | SIB: prostate bed PET uptake 70.2–76.0 Gy; upper pelvis/pelvic nodes 54.0–56.0 Gy (dose tailored to meet OAR constraints) | NR | OAR constraints: rectum & bladder(-CTV) V40Gy and V65Gy specified; small bowel ≤150 cc at 45 Gy (met for all with pelvic RT); penile bulb V40/V65 collected (no protocol constraint) | ADT intent (enrolled): fluciclovine 42/70 (60.0%); PSMA 43/70 (61.4%) | NR | NR | NR |
| Gunnlaugsson et al., (2022) [17] | Combo: pelvic LNI (ENRT/WPRT) for non-responders + PET-directed boost (LN/local if present) + dose escalation | Salvage/curative intent; intensification triggered by PSA non-response during SRT | All: prostate bed 50 Gy/25 fx first 5 weeks; Responders: +20 Gy/10 fx boost to bed (total 70 Gy/35 fx); Non-responders: PSMA-PET reviewed week 5 then (if no macroscopic recurrence) bed +20 Gy/10 fx (total 70/35) + pelvic nodes 50/25; (if local recurrence) +boost to lesion 6 Gy/3 fx (lesion total 76 Gy/38); (if LN recurrence) +boost to LN 10 Gy/5 fx (LN total 60 Gy/30) | NR | VMAT | NR | Prostate bed: 70 Gy/35 fx (50/25 + 20/10) | Pelvic elective (non-responders): 50 Gy/25 fx | LN boost: +10 Gy/5 fx (total 60 Gy/30) if LN recurrence; Local lesion boost: +6 Gy/3 fx (lesion total 76 Gy/38) | NR | NR | 0/97 (0%) | NR | NR | NR/none reported |
| Janbain et al., (2024) [18] | PSMA-PET–guided sRT; elective pelvic lymphatics RT 38.4%; irradiation to PET+ pelvic LNs 30.8%; occasional integrated boost local recurrence; ±ADT 31.6% | Salvage RT post-RP | Prostatic fossa; ±elective pelvic lymphatics; PET+ pelvic LN irradiation | NR | IMRT | Y (image-guided); method NR | EQD2 to prostatic fossa: <66 Gy 10.0%; 66–70 53.6%; >70 36.4% (fx schedule NR) | EQD2 to elective pelvic lymphatics: <50 Gy 30.3%; >50 Gy 4.6% (unknown 3.5%) | EQD2 to positive pelvic LNs: <50 1.5%; 50–60 13.5%; >60 12.4% (unknown 2.4%) | NR | NR | 325/1029 (31.6%) | NR | <6 mo 65 (23.1%); 6–12 110 (39.2%); >12–24 57 (20.3%); >24 49 (17.4%); unknown 44 (4.3%) (as reported) | No escalation beyond ADT reported |
| Jani et al., (2025) [19] | Boost/SIB to PET-uptake (prostate bed up to 76 Gy; pelvis up to 56 Gy); pelvic RT selection based on pelvic nodal uptake/pN+; no RT if extraplelvic/bone uptake | Salvage/curative-intent post-prostatectomy RT (PET-guided) | Prostate bed alone vs prostate bed + whole pelvis (if pelvic LN uptake or pN+); PET-uptake sites received SIB when feasible | Per EMPIRE-1 protocol (general prostate bed and pelvic definitions; PET-to-CT registration for PET-defined targets) | Photon IMRT | Yes; daily IGRT (kV/kV or CBCT) | 64.8–70.2 Gy / 36–39 fx (prostate bed) | 45.0–50.4 Gy / 25–28 fx (pelvic elective, if indicated) | SIB boost: prostate bed uptake to 76.0 Gy (median delivered 74.0 Gy); pelvic uptake to 56.0 Gy (median delivered 55.0 Gy) | NR | PET-defined CTV expanded typically ~0.8 cm while respecting OARs; other OAR constraints NR | Any ADT: Arm 1 42/70 (60%); Arm 2 44/70 (63%); long-term ADT: 14/70 (20%) vs 11/70 (16%) | NR | Typically 6 months (started concurrent with RT); long-term ADT in subset | NR |
| Kirste et al., (2021) [20] | Combo: PET-directed RT (MDT/PDRT to all PSMA+ lesions) ± elective RT (prostate bed and/or pelvic/paraaortic lymphatics); dose escalation via sequential boost or SIB; SBRT used in subset | Curative-intent salvage/oligometastatic treatment (includes MDT/PDRT) | PDRT only lesions (n=204) vs PDRT+elective RT (n=190); elective areas: prostate bed 117/190 (61.6%), pelvic lymphatics 163/190 (85.8%), paraaortic lymph nodes 21/190 (11.1%); elective RT volumes: prostate bed only 23, prostate bed+lymphatics 94, lymphatics only 73 | NR (institution-specific practice) | Conventional fractionated 205 (52.0%); conventional with SIB 130 (33.0%); SBRT 38 (9.6%); conventional + SBRT 21 (5.4%); IMRT/VMAT details NR | NR | Elective prostate bed (ePBRT): median 66 Gy (range 47.5–70) in 1.8–2 Gy/fx | Elective pelvic lymphatics: median 47.5 Gy (range 42–56) in 1.8–2 Gy/fx | Prostate bed PET+ lesion: median 71.2 Gy (62.6–83); pelvic LN lesions: 59.4 Gy (46–85); paraaortic LN lesions: 55 Gy (50–99); sequential boost or SIB; SBRT in subset | NR (SBRT used in 38/394; dose schedules not detailed) | NR (dose converted to EQD2, α/β=1.5 Gy mentioned) | Additive ADT: 130/394 (33.0%); no ADT 262/394 (66.5%); unknown 2/394 (0.5%) | NR | NR | NR (prior chemotherapy excluded) |
| Rogowski et al., (2022) [21] |  |  |  |  |  |  |  |  |  |  |  |  |  |  |  |
| Schmidt-Hegemann et al., (2019) [22] | Boost/SIB (fossa or PET+ nodes) + pelvic nodal irradiation for PET+ LNs; ADT initiation (PET+) | Salvage (curative-intent) RT after RP | Prostatic fossa (all but 1); pelvic lymphatic pathways when PET+ pelvic LNs; SIB/sequential boost to PET+ fossa recurrence and PET+ nodes | RTOG atlas for post-op prostate bed and pelvic LN delineation | IMRT/VMAT | Y (IGRT 2–5×/week) | Prostatic fossa median 66 Gy (59.4–70.2) in 2.0 Gy fractions | Pelvic lymphatic pathways 45–50.4 Gy (median 50.4) | PET+ fossa recurrence 70.0 Gy (67.2–72) via SIB (2.1–2.4 Gy/fx) or sequential boost; PET+ LN 60.76 Gy (54–66) via SIB (1.9–2.4 Gy/fx) or sequential boost (9–12.6 Gy) | NR | NR | ADT 26/90 (28.9%); PET+ 25/42 (60%); 4 ongoing at last follow-up | ADT/antiandrogen (agents NR) | Median 5 mo (range 2–23) among those who stopped; intended 2 y recommended for PET+ | None reported |
| Spohn et al., (2022) [23] | Combo: PET-positive lesion dose escalation (boost) + pelvic elective nodes when nodal disease (ENRT/WPRT strategy); IMRT | Salvage radiotherapy (curative intent) | Prostatic fossa always treated; elective lymphatics/pelvic nodes in case of nodal disease; fossa not omitted even if NR only | Center-specific (standards of care at time); salvage RT concepts referenced in supplementary Table 1 (not detailed in main text) | IMRT (intensity-modulated sRT) | NR | Prostatic fossa/local recurrence dose categories (α/β=1.6): <70 Gy 69%; ≥70 Gy 14%; ≥72 Gy 16% (fx NR) | NR (elective pelvic RT performed in 60%, dose/fx not stated) | NR (dose escalation to PET-positive LR/NR stated; specific boost dose/fx not reported) | NR | NR | ADT 120/235 (51%) | NR | Among ADT: >12 months 49/120 (41%); ≤12 months 71/120 (59%) | NR |
| Tamihardja et al., (2022) [24] | Boost/SIB dose escalation to PET+ local recurrence; SIB boost to PET+ pelvic nodes (if present) | Curative-intent salvage RT | Prostate bed PTV + SIB to macroscopic recurrence; if LN+, additional SIB boost to pelvic LN metastases; elective pelvic nodal irradiation not described | RTOG salvage atlas; adjusted for institutional SIB concept | IMRT or VMAT | Yes; cone-beam CT–guided | 56.1 Gy in 33 fx (1.7 Gy/fx) | NR | Lesion boost: 69.3 Gy (Boost1) in 33 fx (2.1 Gy/fx); subset Boost2 72.6 Gy (IQR 72.6–75.5) in 33 fx (2.2 Gy/fx). LN boost: 66.9 Gy (IQR 61.4–69.3) via SIB | NR | NR | 19 (32.2%) | NR | 24.2 months (IQR 15.4–31.0) | NR |
| Trapp et al., (2024) [25] | ENRT/WPRT field extent comparison (WPRT vs HPRT) with frequent nodal boost | Salvage/curative-intent pelvic nodal RT after RP | Pelvic lymphatic pathways: whole pelvis vs unilateral hemi pelvis; prostate bed RT yes 40/51 (78%) vs 40/51 (85%) | NR (no standardized contouring templates) | NR | NR | NR (prostate bed dose reported only as EQD2 <66 vs ≥66 in Cox model; exact schedules NR) | Pelvic lymphatic pathways dose (EQD2α/β=1.5): ≤50 Gy 42 (82%) vs 44 (86%); >50 Gy 7 (14%) vs 7 (14%); missing 4% vs 0% | LNM boost: yes 46/51 (90%) vs 44/51 (86%). RT dose to LNM (EQD2α/β=1.5): ≤50 Gy 1 (2%) vs 0; 50.1–60 Gy 12 (24%) vs 44 (86%); >60 Gy 30 (59%) vs 0; missing 16% vs 14% | NR | NR | ADT during RT: 35/51 (69%) vs 36/51 (71%) | NR | ADT duration: ≤6 mo 4 (8%) vs 2 (4%); >6–≤12 mo 5 (10%) vs 8 (16%); >12–≤24 mo 5 (10%) vs 2 (4%); >24 mo 1 (2%) vs 3 (6%); missing 36 (71%) vs 36 (71%) | NR |
| Fuertes Vallés et al., (2025) [26] | Combo: focal dose escalation (HDR boost) + ENRT/WPRT component (elective pelvic nodes) + prostate-bed RT | Salvage/curative intent; no MDT/SBRT (HDR boost instead) | HDR: GTV (IPBR) + 5-mm margin CTV; EBRT: PTV2 prostate & seminal vesicle bed; PTV3 elective pelvic nodes (external/internal iliac, obturator) | Institutional protocol (described target definitions) | EBRT: VMAT or IMRT; HDR brachytherapy under TRUS with MRI/TRUS fusion + intraoperative MRI | Yes; daily cone-beam CT; fiducials | 42 Gy / 15 fx (PTV2) | 40 Gy / 15 fx (PTV3 elective pelvic nodes) | HDR boost: 19 Gy in 2 fractions to CTV (two consecutive days); (not SIB) | NR | Example constraints: rectum 2cc <5.7 Gy/fx; VUan D50 ipsi <6.0 Gy/contra <4.0; EUS D50 ipsi <5.0/contra <3.0; bladder neck 2cc <6.6 Gy/fx; cumulative EQD2 constraints also specified | 16 (100%) | LHRH agonist; antiandrogen for 10 days prior to LHRH initiation | 6 | None reported |
| Vogel et al., (2021) [27] | Boost/SIB (dose escalation) to PSMA PET–positive lesion(s) within salvage RT; may include elective nodal irradiation | Salvage/curative-intent | C-SRT: PB 85/98 (86.7%); PB+ePLNs 13/98 (13.3%). DE-SRT: PB/SIB 55/101 (54.5%); PB/SIB+ePLNs 11/101 (10.9%); PB+ePLNs/SIB 16/101 (15.8%); PB/SIB+ePLNs/SIB 15/101 (14.8%); ePLNs/SIB 4/101 (4.0%) | Target delineation followed RTOG or EORTC recommendations | IMRT (VMAT or helical IMRT) | IGRT with daily online imaging; planning/RT with full bladder and empty rectum | Prostate bed: 68.0 Gy total; 2.0 Gy/fx | Elective pelvic LNs (when treated): 50.4 Gy total; 1.8 Gy/fx | PET+ LN SIB: 58.8 Gy (58.8–61.6); 2.10 Gy/fx (1.80–2.25). PET+ local recurrence SIB: 76.5 Gy (73.10–76.50); 2.25 Gy/fx (2.00–2.25) | NR | PTV margin for SIB: 5–10 mm | Additive ADT: 40/199 (20.1%); C-SRT 12/98 (12.2%); DE-SRT 28/101 (27.7%) | NR | NR | NR |
| ENRT, elective nodal radiotherapy; LN, lymph node; LND, lymph node dissection; LVI, lymphovascular invasion; MDT, metastasis-directed therapy; miTNM, molecular imaging TNM; NR, not reported; pN, pathological nodal stage; pT, pathological tumor stage; PRO, patient-reported outcome; QoL, quality of life; ¹⁸F, fluorine-18; ⁶⁸Ga, gallium-68. | | | | | | | | | | | | | | | |
