# Supplementary material for: PSMA PET–guided intensification of postprostatectomy salvage radiotherapy for prostate cancer: a systematic review and meta-analysis
Source: Front Oncol. 2026 Mar 10;16:1779689. doi: 10.3389/fonc.2026.1779689 (PMC13008707; doi:10.3389/fonc.2026.1779689)
Supplement: Supplementary file 6 [file Table4.docx]

| Supplementary Table S1D. Outcomes, toxicity, and QoL | | | | | | | | | | | | | | | | | | | | |
| --- | --- | --- | --- | --- | --- | --- | --- | --- | --- | --- | --- | --- | --- | --- | --- | --- | --- | --- | --- | --- |
| Study (year) | Outcome definition: FFS/bRFS (exact) | Effect size (HR) for FFS/bRFS | 95% CI for HR | Time-point bRFS/FFS (2y) | Time-point bRFS/FFS (3y) | Time-point bRFS/FFS (5y) | Metastasis outcome definition (MFS/DMFS) | Effect size (HR) for MFS/DMFS | Time-point MFS/DMFS (2y/3y/5y) | OS/PCSS outcomes | Acute toxicity: GU ≥G2 (n/N; by arm if comparative) | Acute toxicity: GI ≥G2 (n/N; by arm) | Acute toxicity: GU ≥G3 (n/N; by arm) | Acute toxicity: GI ≥G3 (n/N; by arm) | Late toxicity: GU ≥G2 (n/N; by arm) | Late toxicity: GI ≥G2 (n/N; by arm) | Late toxicity: GU ≥G3 (n/N; by arm) | Late toxicity: GI ≥G3 (n/N; by arm) | QoL / PRO instrument | QoL results |
| Arifin et al., (2023) [14] | BFFS definition: time from salvage RT to biochemical failure, death, or last PSA; BF defined by rising PSA requiring salvage systemic therapy (exact numeric threshold NR) | Cox HR for BFFS (matched, univariable): 1.00 | 0.32–3.10 | 2y BFFS (matched): 76.0% vs 73.0% | NR | NR | MFS definition: time from salvage RT to metastatic disease (M1 on conventional or PSMA imaging) or death | Cox HR for MFS (all, univariable): 0.19 (adjusted: 0.24) | Matched 2y MFS: 97.0% vs 84.9% (p=0.046) | OS not analyzed (no deaths reported) | NR | NR | NR | NR | NR | NR | NR | NR | NR | NR |
| Petit et al ., (2025) [7] | FFS: time from randomization to biochemical failure, treatment failure (palliative systemic therapy), or radiographic progression. Biochemical failure: PSA nadir >0.2 ng/mL OR rise to a second value ≥0.2 ng/mL and ≥0.05 ng/mL higher than prior value | HR 0.50 (primary analysis, PSMA-PET–guided vs standard) | 0.27–0.94 (P=0.04) | NR | NR | NR | Not separately reported; radiographic progression included in FFS definition | NR | NR | NR | NR (GU ≥G2 not separately reported; detailed breakdown referenced to Supplement 2 eTable 4) | NR (GI ≥G2 not separately reported) | NR (GU ≥G3 not separately reported; overall grade ≥3 RT-related events 3/64 vs 6/64) | NR (GI ≥G3 not separately reported) | NR | NR | NR | NR | EPIC-CP; FACT-P; IPSS | No clinically meaningful between-group QoL differences reported; questionnaire completion ~90%+ at baseline and end-of-RT (numeric domain results NR) |
| Bluemel et al., (2016) [15] | NR (no formal FFS/bRFS; biochemical response assessed via PSA decline) | NR | NR | NR | NR | NR | NR | NR | NR | NR | NR | GI ≥G2: 2/36 (5.6%) during SRT (1 prostate-bed SRT; 1 with presacral LNM boost) | NR | NR (no ≥G3 events reported) | NR | NR | NR | NR | NR | NR |
| Dhere et al., (2025) [16] | NR (no FFS/bRFS definition reported in this acute-toxicity analysis; biochemical control forthcoming) | NR | NR | NR | NR | NR | NR | NR | NR | NR | Acute GU ≥G2: fluciclovine 10/59 (16.95%); PSMA 4/60 (6.67%) | Acute GI ≥G2: fluciclovine 3/59 (5.08%); PSMA 1/60 (1.67%) | Acute GU ≥G3: 0/59 vs 0/60 | Acute GI ≥G3: 0/59 vs 0/60 | NR | NR | NR | NR | NR (patient-reported outcomes forthcoming) | NR |
| Gunnlaugsson et al., (2022) [17] | Treatment failure = BCR or clinical recurrence; BCR defined as PSA rise ≥0.2 ng/mL above nadir confirmed by another PSA | NR | NR | NR | FFS(3y): overall 76%; responders 94%; non-responders 68% | NR | NR | NR | NR | NR | NR | End of RT: Resp 5/34 (15%); Non-resp 9/63 (14%) | End of RT: Resp 4/34 (12%); Non-resp 17/63 (27%) | NR | NR | 12 mo: Resp 3/33 (9%); Non-resp 1/59 (2%) | 12 mo: Resp 4/33 (12%); Non-resp 10/59 (17%) | NR | NR | EORTC QLQ-PR25 (urinary & bowel symptom scales) |
| Janbain et al., (2024) [18] | FFBF: 2 consecutive rising PSA values >0.2 ng/mL after treatment | NR (ML model; no HR) | NR | NR | NR | NR | NR | NR | NR | NR | NR | NR | NR | NR | NR | NR | NR | NR | NR | NR |
| Jani et al., (2025) [19] | EFS (from end of RT): PSA >0.2 ng/mL from nadir followed by another rise; OR persistent PSA; OR imaging/DRE failure; OR initiation of systemic therapy | NR (HR not reported in main text for arm comparison; proportions/log-rank used) | NR | 2-yr EFS: Arm 2 (PSMA) 88% (7 events; RT completers n=60); Arm 1 (fluciclovine) 87% (7 events; n=59) | NR | NR | NR | NR | NR | NR | NR | NR | NR | NR | NR | NR | Late GU ≥G3 (urethral stricture): Arm 1 2/59 (3.6%); Arm 2 1/60 (1.6%) | NR | NR | NR |
| Kirste et al., (2021) [20] | bRFS primary; event = PSA increase ≥0.2 ng/mL above nadir after definitive treatment of PSMA-PET recurrences (EAU/ASTRO/AUA); if PSA did not respond to RT, event defined as rise ≥0.2 ng/mL from pre-RT PSA; time-to-event from last day of RT | Elective RT vs no elective RT (multivariable Cox): HR 0.33 | 0.15–0.73 | NR | 3-year bRFS: 37% (PDRT) vs 53% (PDRT+eRT) | NR | NR | NR | NR | NR | PDRT: 2/204; PDRT+eRT: 31/190 | PDRT: 12/204; PDRT+eRT: 44/190 | PDRT: 0/204; PDRT+eRT: 0/190 | PDRT: 0/204; PDRT+eRT: 1/190 | PDRT: 1/204; PDRT+eRT: 42/190 | PDRT: 2/204; PDRT+eRT: 10/190 | PDRT: 0/204; PDRT+eRT: 7/190 | PDRT: 0/204; PDRT+eRT: 0/190 | NR | NR |
| Rogowski et al., (2022) [21] |  |  |  |  |  |  |  |  |  |  |  |  |  |  |  |  |  |  |  |  |
| Schmidt-Hegemann et al., (2019) [22] | BRFS endpoint: PSA ≤0.2 ng/mL (time-to-event); follow-up defined from RT end to last recorded PSA | NR (no comparative HR reported; Cox predictors reported) | NR | NR (reported 78% BRFS at last follow-up; median follow-up 23 mo) | NR | NR | NR (no formal MFS/DMFS endpoint; no distant metastases observed during follow-up) | NR | NR | No deaths; no distant metastases during follow-up | Acute GU ≥G2: 12/90 (13%) | Acute GI ≥G2: 14/90 (16%) | Acute GU ≥G3: 0/90 | Acute GI ≥G3: 0/90 | Late GU ≥G2: 12/90 (13%) | Late GI ≥G2: 3/90 (3%) | Late GU ≥G3: 2/90 (2%) | Late GI ≥G3: 0/90 | NR | NR |
| Spohn et al., (2022) [23] | BRFS: PSA >0.2 ng/mL above post-sRT nadir without additional salvage therapy or death | SUVmax in LR >75% quartile vs <75%: HR 2.3 | 95% CI 1.1–4.6 | 2y BRFS 68% overall; 80% (LR-only); 65% (NR-only) | NR | NR | NR | NR | NR | No patient died during follow-up (OS/PCSS not otherwise reported) | NR | NR | NR | NR | NR | NR | NR | NR | NR | NR |
| Tamihardja et al., (2022) [24] | Biochemical progression-free survival; biochemical progression = PSA increase ≥0.5 ng/mL over PSA nadir (GETUG AFU 16) | NR (no comparator); prognostic Cox models reported for covariates | NR | NR | 89.1% (95% CI 81.1–97.8) at 3 years | NR | MFS = time from start of RT to imaging-based distant metastasis | NR (no comparator); prognostic multivariable HRs reported (e.g., GS≥8, time RP→SRT) | 96.2% (95% CI 91.2–100.0) at 3 years; 2y/5y NR | 3-year OS 100%; 2 deaths (3.4%) during follow-up; PCSS NR | 13/59 (22.0%) | 3/59 (5.1%) | 1/59 (1.7%) | 0/59 (0%) | 12/59 (20.3%) cumulative at 3 years | 1/59 (1.7%) | 2/59 (3.4%) cumulative at 3 years (urinary incontinence) | 0/59 (0%) | NR | NR |
| Trapp et al., (2024) [25] | BRFS endpoint: biochemical recurrence defined as PSA rise ≥0.2 ng/mL above post-RT nadir | NR (no HR for WPRT vs HPRT reported; log-rank p=.97) | NR | 2y BRFS: 61% (WPRT) vs 57% (HPRT) | NR | NR | MFS definition: metastases defined as hematogenous metastases or supradiaphragmatic LNM | NR | 2y MFS: 86% (WPRT) vs 90% (HPRT) | NR | NR (toxicity not available in dataset) | NR | NR | NR | NR | NR | NR | NR | NR | NR |
| Fuertes Vallés et al., (2025) [26] | Primary endpoint: biochemical relapse-free survival (bRFS) at 5 years | NR (single-arm; no HR) | NR | NR | NR (reported 15/16 biochemically controlled at median 3.7y) | 92.7% (KM estimate); 15/16 (93.8%) biochemically controlled at median 3.7y; no local failures | NR | NR | NR (1 patient para-aortic nodal relapse at 2.7y) | NR | NR | NR | NR | NR | 1/16 (6.3%) | 0/16 | 0/16 | 0/16 | EORTC QLQ-PR25 | Urinary symptom score mean 5→7 at 6 months; functional scores 52→55 (minor/non-significant changes) |
| Vogel et al., (2021) [27] | Primary oncologic endpoint reported as DFS (FFS-like): event = PSA progression (PSA nadir+0.2 confirmed) and/or local relapse and/or metastasis and/or initiation/change of ADT | HR 2.22 (Figure 1; Cox regression adjusted for ADT; interpret direction during synthesis) | 1.02–4.82 | NR | NR | NR | MFS/DMFS not reported | NR | NR | OS/PCSS not reported | Acute GU ≥G2: C-SRT 2.1% vs DE-SRT 3.0% (analysis subset n=95 vs 99) | Acute GI ≥G2: C-SRT 2.1% vs DE-SRT 1.0% (n=95 vs 99) | Acute GU ≥G3: C-SRT 0% vs DE-SRT 1.0% (n=95 vs 99) | Acute GI ≥G3: C-SRT 1.1% vs DE-SRT 0% (n=95 vs 99) | Late GU ≥G2: C-SRT 11.0% vs DE-SRT 14.7% (n=73 vs 75) | Late GI ≥G2: C-SRT 1.4% vs DE-SRT 5.3% (n=73 vs 75) | Late GU ≥G3: C-SRT 1.4% vs DE-SRT 5.3% (n=73 vs 75) | Late GI ≥G3: C-SRT 0% vs DE-SRT 2.7% (n=73 vs 75) | PSA response defined as PSA ≤0.2 ng/mL; PSA response at first follow-up: 57.5% (C-SRT) vs 75.0% (DE-SRT); at last follow-up: 69.6% vs 86.4% | Include (comparative PSMA PET–guided intensification with SIB/ENRT elements; provides DFS and toxicity) |
| ENRT, elective nodal radiotherapy; LN, lymph node; LND, lymph node dissection; LVI, lymphovascular invasion; MDT, metastasis-directed therapy; miTNM, molecular imaging TNM; NR, not reported; pN, pathological nodal stage; pT, pathological tumor stage; PRO, patient-reported outcome; QoL, quality of life; ¹⁸F, fluorine-18; ⁶⁸Ga, gallium-68. | | | | | | | | | | | | | | | | | | | | |
